# Titin governs myocardial passive stiffness with major support from microtubules and actin and the extracellular matrix

**DOI:** 10.1038/s44161-023-00348-1

**Published:** 2023-10-26

**Authors:** Christine M. Loescher, Johanna K. Freundt, Andreas Unger, Anthony L. Hessel, Michel Kühn, Franziska Koser, Wolfgang A. Linke

**Affiliations:** Institute of Physiology II, University of Muenster, Muenster, Germany

**Keywords:** Cardiovascular biology, Biophysics, Cell biology

## Abstract

Myocardial passive stiffness is crucial for the heart’s pump function and is determined by mechanical elements, including the extracellular matrix and cytoskeletal filaments; however, their individual contributions are controversially discussed and difficult to quantify. In this study, we targeted the cytoskeletal filaments in a mouse model, which enables the specific, acute and complete cleavage of the sarcomeric titin springs. We show in vitro that each cytoskeletal filament’s stiffness contribution varies depending on whether the elastic or the viscous forces are considered and on strain level. Titin governs myocardial elastic forces, with the largest contribution provided at both low and high strain. Viscous force contributions are more uniformly distributed among the microtubules, titin and actin. The extracellular matrix contributes at high strain. The remaining forces after total target element disruption are likely derived from desmin filaments. Our findings answer longstanding questions about cardiac mechanical architecture and allow better targeting of passive myocardial stiffness in heart failure.

## Abstract (web summary)

Loescher, Freundt et al. investigated the contribution of each cytoskeletal filament to passive myocardial stiffness by using a mouse model that allows for specific cleavage of titin; they show that titin is a major cytoskeletal filament controlling myocardial elastic forces, whereas viscous forces are controlled by titin, the microtubules and actin.

## Main

Dynamic regulation of myocardial passive stiffness is crucial for regulating cardiac output, as is well described by the Frank–Starling mechanism. However, increased myocardial stiffening is also a major characteristic of heart failure (HF), especially HF with preserved ejection fraction (HFpEF; refs. ^1–3^). Myocardial stiffness is derived from numerous protein networks and filament systems, including the extracellular matrix (ECM), the microtubule (MT) network, titin, the microfilament (actin) network and the intermediate filament (IF) network^4–8^. Many of these networks have been shown to alter their stiffness properties under both physiological and pathological conditions^4–7^. In addition, the cell membrane (sarcolemma) provides the containment lines that all the aforementioned networks need to work within or around, but its direct stiffness contribution remains unclear^9^. In line with the Frank–Starling mechanism, the contribution of any network or filament system to overall stiffness will also depend on cardiac filling and associated myocardial strain^1^. Moreover, all these networks, particularly within the cardiomyocytes themselves, are inherently intertwined to form the structural framework for the cell^10^. Therefore, removing one network may directly impact the tensional integrity and passive force contribution of another, a prime example being interactions between the sarcomeric proteins titin and actin^11,12^.

Although myocardial passive stiffness properties of mechanical network members have been investigated individually by acutely disrupting them pharmacologically^12,13^, this approach has not been possible to specifically quantify the contribution of titin. Titin is considered one of the most critical proteins in regulating overall myocardial stiffness, with titin isoform switching, changes in post-translational modifications and haploinsufficiency^6,7,14^ all having been implicated in myocardial passive stiffness changes and HF. However, specifically targeting titin to directly quantify its contribution to myocardial stiffness has not been possible. We overcome these obstacles using our recent genetic mouse model, the titin cleavage (TC)-Halo mouse, which allows us to acutely, specifically and completely sever titin within the spring region^15–17^. The insertion of the genetic cassette causes no pathological effects, with the mice having normal heart and muscle function, and no alterations are seen in the ultrastructure of the sarcomeres^15,16^. The TC-Halo mouse has provided critical information on the mechanical role of titin in skeletal muscle but has not yet been used to investigate passive stiffness properties of the myocardium^15–17^.

A further challenge is that there are various ways to define and quantify myocardial stiffness, making it difficult to compare myocardial stiffness contributions of individual mechanical network members obtained from different studies^3^. In this study, we focused on uniaxial tensile passive stiffness/forces of the left ventricular (LV) myocardium. Moreover, we considered the viscoelastic properties of the myocardium in more depth by evaluating the velocity-insensitive (‘elastic’) component and the velocity-sensitive (‘viscous’) component of the myocardial tensile passive forces separately.

Using the TC-Halo mouse, we systematically ‘play musical chairs’ with the main contributors to myocardial passive stiffness—the MT network, sarcolemma, titin and actin—by removing them one by one. The contribution of the ECM is assessed by comparing LV fiber bundles and individual cardiomyocytes, and we infer the role of the IFs/desmin through the extensive elimination procedures used. Moreover, we explore the tensegral relationship between titin and actin by looking at disruption history-dependent effects and reciprocal interactions. We found that titin is the largest contributor to elastic passive forces of mouse myocardium, whereas the viscous component is mostly a combination of MT-based, titin-based and actin-based forces. The distribution of passive stiffness among the structural elements is also dependent on fiber bundle strain, with the ECM becoming highly relevant for the elastic and viscous forces at higher strain. Our data provide not only a precise quantification of the individual contributions from the different structural elements to myocardial stiffness in a single model but also much-needed information to guide therapeutic strategies aiming to target the pathological stiffness of the diseased heart, such as in HFpEF.

## Results

The structural integrity of the myocardial wall can be attributed to several mechanical elements, including MTs, sarcolemma, titin, actin, ECM and IFs. Taking a systematic approach, we compared the respective contributions of these elements to the passive elastic and viscous forces of mouse LV myocardium over a range of fiber bundle strains and also visualized the substructure of the heart before and after the acute removal of each of these components.

### MTs substantially contribute to viscous forces

**Fig. 1:**
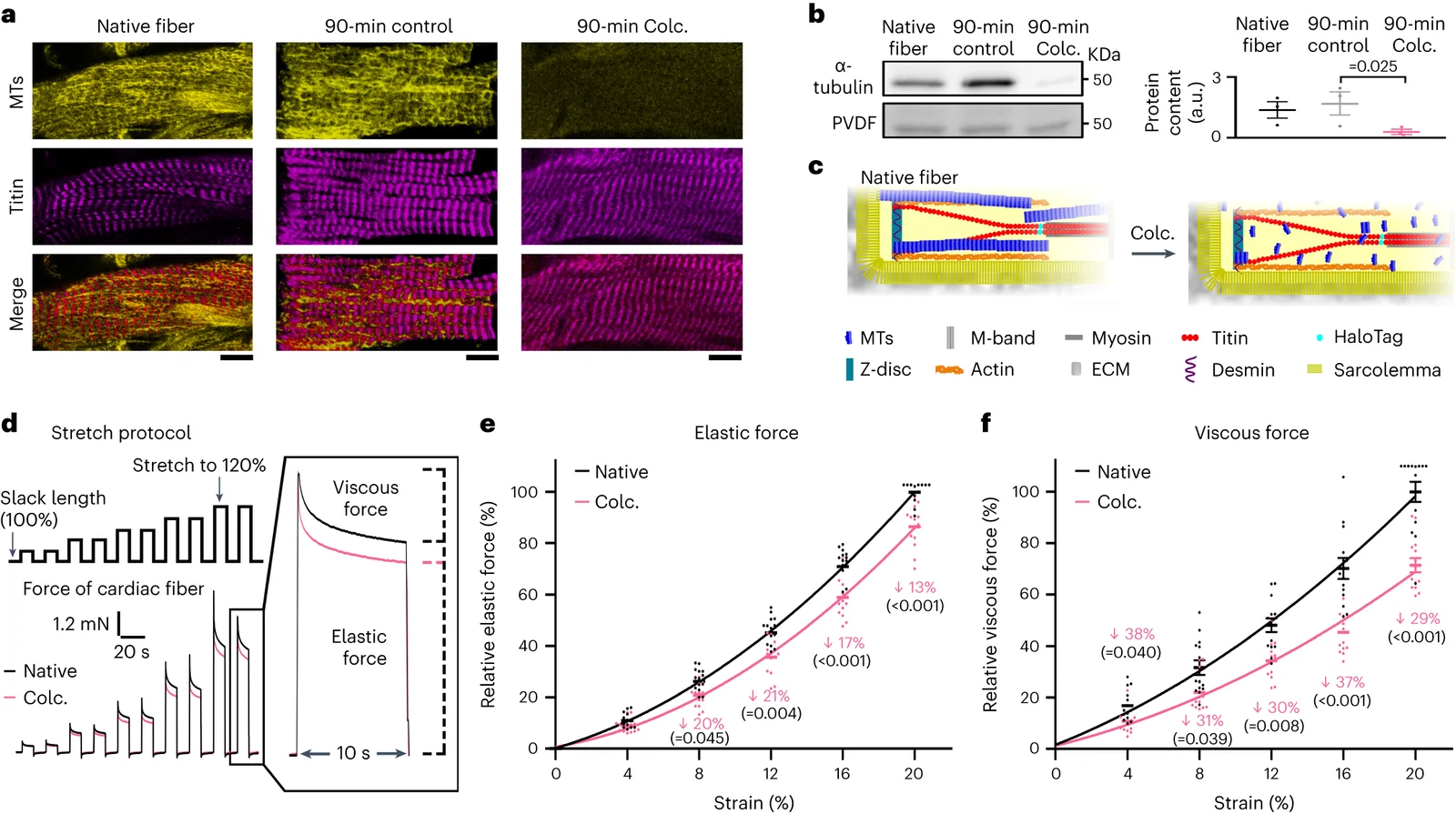
MT network structure and passive force contributions in cardiac LV fiber bundles. **a**, Confocal images of native cardiac fiber bundles fixed immediately after isolation, perfused in NT buffer for 90 min as a control or treated with 30 µM colchicine (Colc.) for 90 min. The MTs were stained with anti-α-tubulin (top; secondary antibody, Alexa Fluor 488-conjugated IgG) and titin with α-TTN5 (middle; secondary antibody, Alexa Fluor Cy3-conjugated IgG); a merge image was added (bottom). Scale bars, 10 µm. Similar findings were obtained from *N* = 3 mice per group from *n* = 30 images. **b**, Matched samples from **a** were also used to detect the polymerized fraction of the MTs for western blot against α-tubulin with Coomassie staining of the PVDF membrane used as loading control, for quantification (*n* = 3). **c**, Pictographic representation of the Colc. treatment effect. **d**, Stretch protocol performed on cardiac fiber bundles and example traces from a cardiac fiber bundle measured under native conditions and after a 90-min incubation with 30 µM Colc. Inset is an enlargement of the 20% strain trace indicating the passive force components analyzed (elastic and viscous). Elastic (**e**) and viscous (**f**) forces under native conditions followed by Colc. treatment (*N* = 7 and *n* = 14). Forces are relative to the highest elastic/viscous force measured at 20% strain under native conditions. The significant force reduction after Colc. for a given strain is stated (pink values). All data are mean ± s.e.m. *N* refers to the number of animals used, and *n* refers to the number of fibers measured (**b**) or the number of measurements made for each strain level, including the two technical replicates measured for each cardiac fiber bundle (**e**,**f**). Significance (*P* values stated in black) was determined using a Kruskal–Wallis test and Dunn’s test (**b**) or two-way repeated-measures ANOVA followed by Sidak’s multiple comparisons test (**e**,**f**). Curves were fitted with a second-order polynomial (**e**,**f**). Source data

Starting with native cardiac LV fiber bundles, we first targeted the extensive MT network. We confirmed with both confocal imaging (Fig. 1a) and western blot (Fig. 1b) that 90-min incubation with colchicine caused the complete disruption of the MT network (Fig. 1c).

To measure the passive force contribution of the MTs, we performed a stepwise stretch protocol to extend the native LV fiber bundles from slack length (0% strain) to 20% strain (Fig. 1d). These strain levels approximate a range of physiological cardiomyocyte strains from low to high. For each step, the fiber bundle was stretched to a given strain level and held for 10 s before being released to its starting length (0% strain). The measured forces for each step were divided into the velocity-insensitive elastic component by fitting the 10-s force relaxation curve to determine the steady state and the velocity-sensitive viscous component by comparing the difference between the peak force after stretch and the calculated steady state (Fig. 1d, inset). We then disrupted the MT network using a 90-min colchicine treatment and compared the passive forces before and after the disruption. The difference in force elicited was taken as the force contribution of the MTs for each fiber bundle strain.

**Extended Data Fig. 1:**
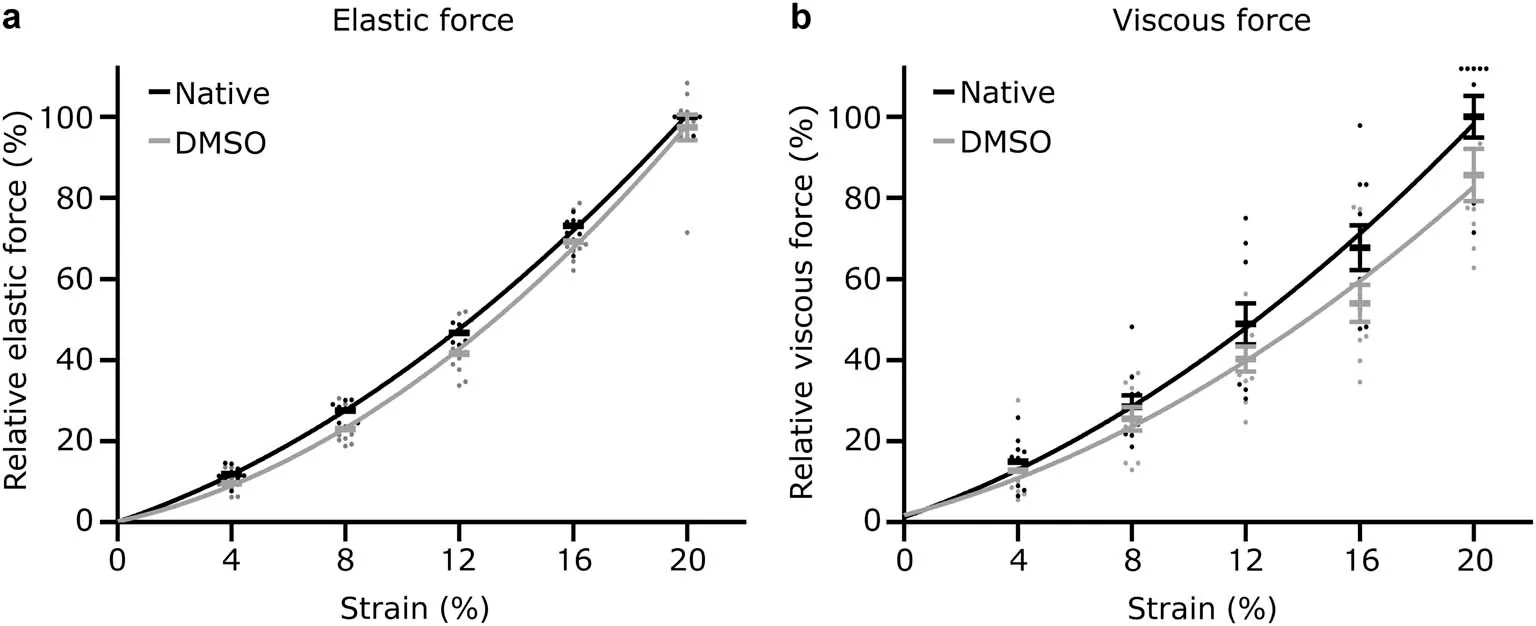
Passive forces after 90-min incubation with DMSO. (a) Elastic and (b) viscous forces under native conditions followed by a 90-min DMSO control incubation. Forces are relative to the elastic and viscous forces measured at 20% strain under native conditions. Data expressed as mean ± SEM, N = 5, n = 10. N refers to the number of animals used and n refers to the number of measurements made for each strain level including the two technical replicates measured for each cardiac fiber bundle. No important differences were found using a 2-way repeated measures ANOVA followed by a Sidak’s multiple comparisons test. Curves fitted with a 2nd order polynomial. Source data

The disruption of the MT network caused a significant reduction in elastic force (Fig. 1e). The contribution to total elastic force, relative to the native fiber bundle, decreased from 22% at 8% strain to only 14% at 20% strain (Fig. 1e). The viscous force contribution of the MTs was relatively higher than the elastic force contribution overall but also became smaller with increasing strain (36% contribution at 4% strain and 29% contribution at 20% strain) (Fig. 1f). A 90-min incubation with DMSO only (control) had no impact on the MT network’s contribution to passive forces (Extended Data Fig. 1).

### The sarcolemma has low relevance for the viscoelastic forces

**Fig. 2:**
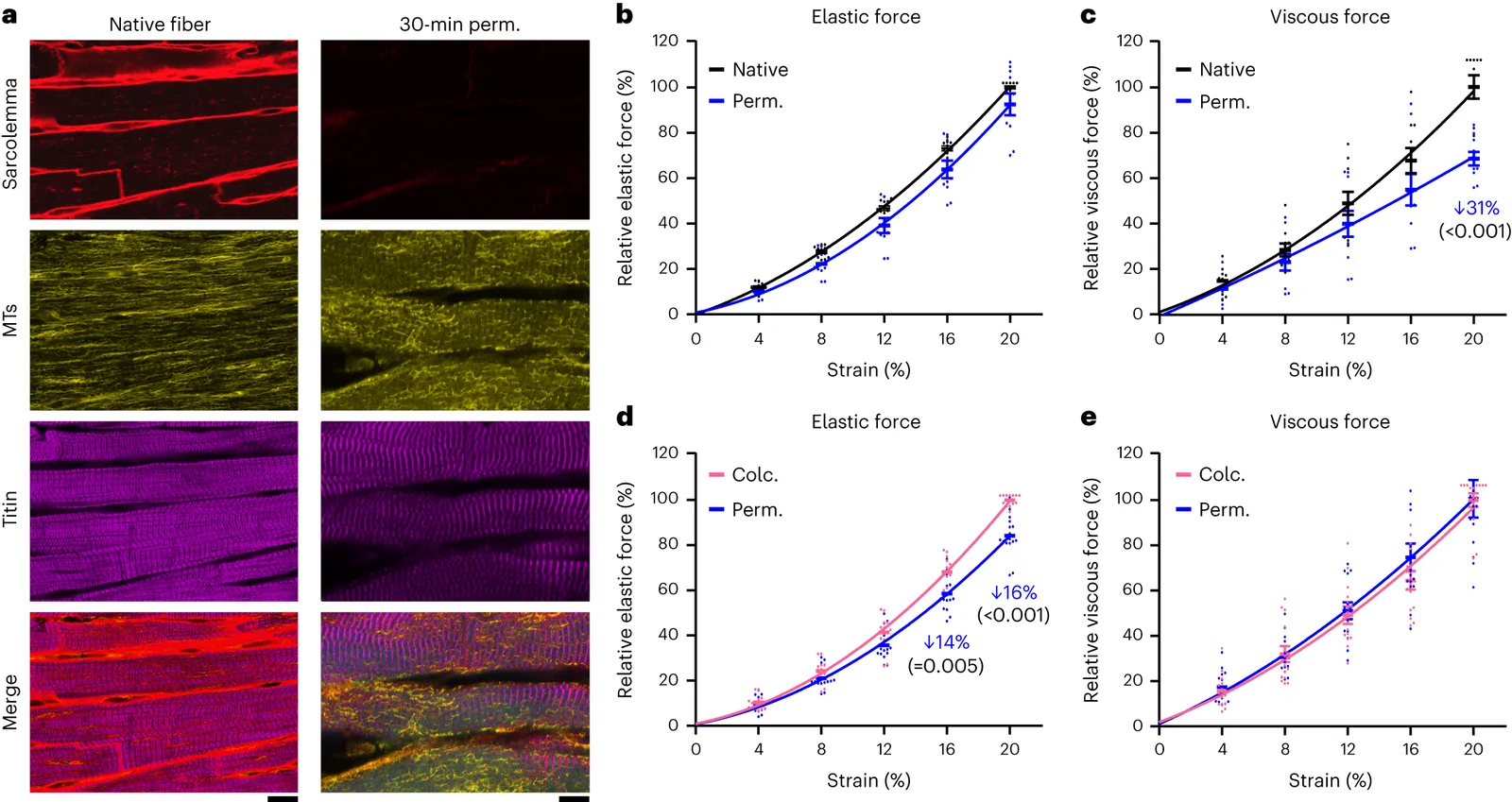
Sarcolemma constitution and passive force contribution in cardiac LV fiber bundles. **a**, Confocal images of an LV fiber bundle area in native state and after permeabilization for 30 min with Triton X-100. Sarcolemma was stained with wheat germ agglutinin, MTs with anti-α-tubulin (secondary antibody, Alexa Fluor 488-conjugated IgG), titin with anti-HaloTag (secondary antibody, Alexa Fluor 647-conjugated IgG) and merge (bottom). Scale bars, 10 µm. Similar findings were obtained from *N* = 5 mice per group from *n* = 40 images. Elastic (**b**) and viscous (**c**) forces of native cardiac fiber bundles (Native) and then permeabilized for 30 min with Triton X-100 (Perm., *N* = 5, *n* = 10). Elastic (**d**) and viscous (**e**) forces after colchicine (Colc.) treatment first and then 30-min permeabilization (Perm.) with Triton X-100 (*N* = 7, *n* = 14). Forces are relative to the mean elastic and viscous forces measured at 20% strain in native fiber bundles (**b**,**c**) or after Colc. treatment (**d**,**e**). The significant force reduction after Perm. for a given strain is stated (blue values). Data are mean ± s.e.m. *N* refers to the number of animals used, and *n* refers to the number of measurements made for each strain level, including the two technical replicates measured for each cardiac fiber bundle. Significance was determined using a two-way repeated-measures ANOVA followed by Sidak’s multiple comparisons test (*P* values stated in black). Curves were fitted with a second-order polynomial. Source data

**Extended Data Fig. 2:**
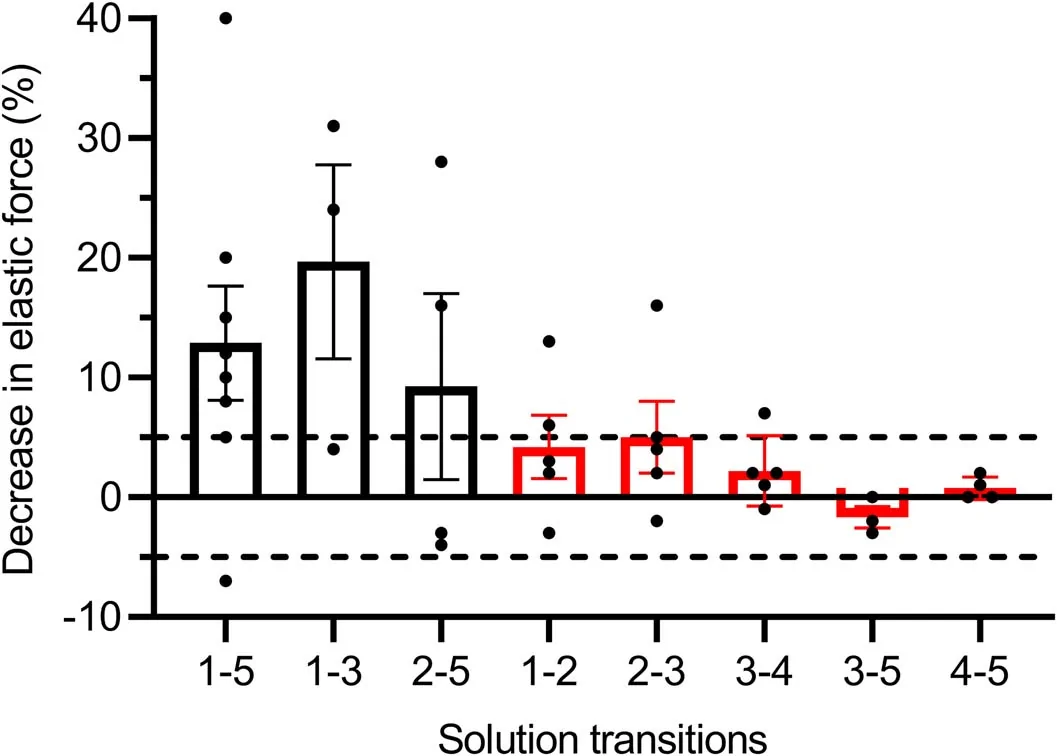
Transitioning between different solution compositions can contribute to passive forces. The removal of the sarcolemma required consideration for the movement between an extracellular (Na^+^ rich) environment to an intracellular environment (K^+^ rich) and differing Ca^2+^ conditions. Additional care needed to be taken to ensure passive force was not generated through the solution transitions alone. Numerous solution transitions were tested to determine how to move from the extracellular normal Tyrode’s (NT) solution to the intracellular relaxing solution (RS) and stay within the acceptable ±5% passive force change limit (dashed lines). Final solution transitions used are marked in red, data expressed as mean ± SEM, n is the number of technical replicates (n = 3 for solution transitions 1-3 and 3-5, n = 4 for solution transitions 2-5 and 4-5, n = 5 for solution transitions 1-2, 2-3 and 3-4, and n = 8 for solution transition 1-5). Solution 1- NT (140 mM NaCl, 0.5 mM MgCl_2_, 0.33 mM NaH_2_PO_4_, 5 mM HEPES, 5.5 mM glucose, 5 mM KCl, adjusted to pH 7.4 with NaOH). Solution 2- NT with 1 mM EGTA added. Solution 3- Sodium based RS (170 mM Na-Propionate, 20 mM MOPS, 2.5 mM Mg-Acetate, 5 mM K_2_EGTA, 2.5 mM ATP, 14.5 mM creatine phosphate, 1x working concentration of protease inhibitor cocktail (Promega: G6521), pH 7.0 at 0 °C. Solution 4- Sodium based RS with an additional 0.5 % Triton X-100. Solution 5- potassium-based RS (170 mM K-Propionate instead of Na-Propionate). Source data

The sarcolemma is critical in separating the extracellular and intracellular environments and provides anchoring points for many structural proteins. However, whether it directly contributes to passive forces in the myocardium is still unclear^9^. Confocal imaging confirmed the complete permeabilization of the sarcolemma with a 30-min treatment using the detergent Triton X-100 (Fig. 2a), but, interestingly, this treatment did not completely disrupt the MT network as expected based on previous reports^18^. When the MT network was still in place during permeabilization, the sarcolemma did not contribute to elastic force at all (Fig. 2b) and only contributed to viscous force at high strain (Fig. 2c). We carefully considered the solution composition during the transition of the fiber bundle from the extracellular to the intracellular environment to ensure that passive force changes were not an artifact of the solution environment (Extended Data Fig. 2).

Additionally, we removed the MTs first (using colchicine) and only then permeabilized the cardiac fiber bundles using 30-min Triton X-100 before repeating the stretch protocol (Fig. 2d,e). In the absence of the MT network, the sarcolemma made a small contribution to elastic forces at larger strains—14% and 16% at 16% and 20% strain, respectively (Fig. 2d)—and did not contribute to viscous forces (Fig. 2e). This finding suggests that the MT network has a direct impact on the apparent passive force contribution of the sarcolemma, and that the latter contributes in a limited manner to passive forces, mainly by helping contain and stabilize the MT network.

### Actin greatly contributes to both elastic and viscous forces

**Fig. 3:**
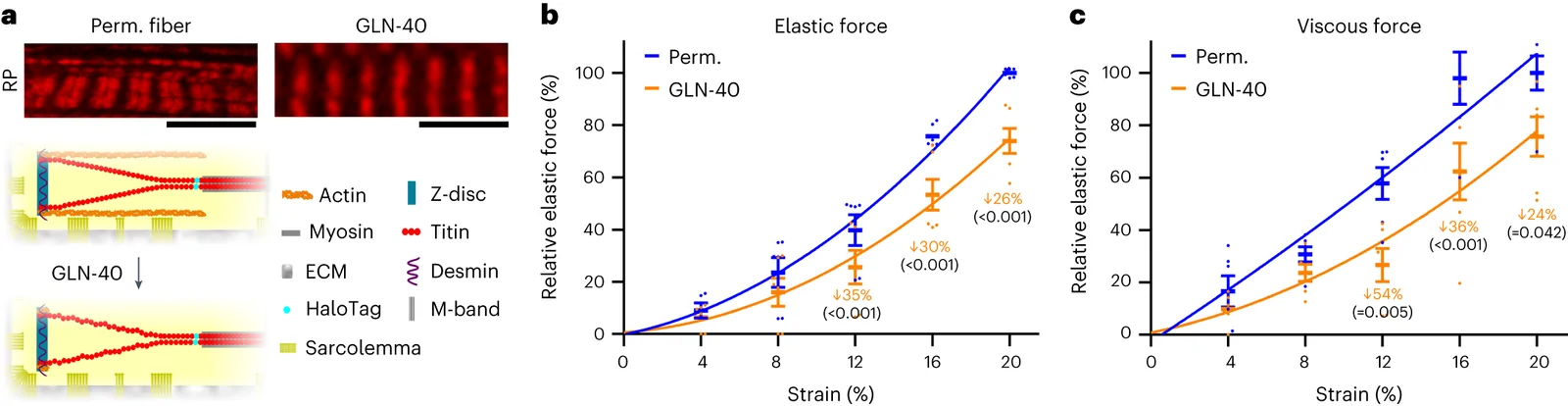
Actin passive force contribution in cardiac LV fiber bundles. **a**, Confocal images of a permeabilized (Perm.) fiber bundle area stained with RP before and after actin extraction with gelsolin (GLN-40, top) and cartoons of the treatment effect (middle and bottom). Scale bars, 5 µm. Similar findings were obtained from *N* = 2 mice from *n* = 10 images. Elastic (**b**) and viscous (**c**) forces of Perm. cardiac fiber bundles before and after actin severing with GLN-40 (*N* = 3, *n* = 6). Forces are relative to the highest elastic and viscous forces measured at 20% strain of the Perm. fiber bundle before actin extraction. The significant force reduction after GLN-40 versus Perm. for a given strain is stated (orange values). Data are mean ± s.e.m. *N* refers to the number of animals used, and *n* refers to the number of measurements made for each strain level, including the two technical replicates measured for each cardiac fiber bundle. Significance was determined using a two-way repeated-measures ANOVA followed by Sidak’s multiple comparisons test (*P* values stated in black). Curves were fitted with a second-order polynomial. Source data

With the MT network and the sarcolemma removed, we next wanted to determine the contribution of the actin network to passive force. Staining the LV tissue with rhodamine-phalloidin (RP) highlighted the extensive network of intra-sarcomeric and extra-sarcomeric actin, which we could then sever using a Ca^2+^-independent fragment of gelsolin (GLN-40; ref. ^12^). This treatment left only a small fragment of F-actin within the Z-discs of the sarcomeres (Fig. 3a).

The severing of actin resulted in ~30% significant decreases in elastic force at mid to high strain (12–20% strain; Fig. 3b). Similarly, a significant decrease in viscous force of 54%, 36% and 24% was detected at 12%, 16% and 20% strain, respectively (Fig. 3c).

### Titin is acutely cleaved in TC-Halo mouse heart tissues

**Fig. 4:**
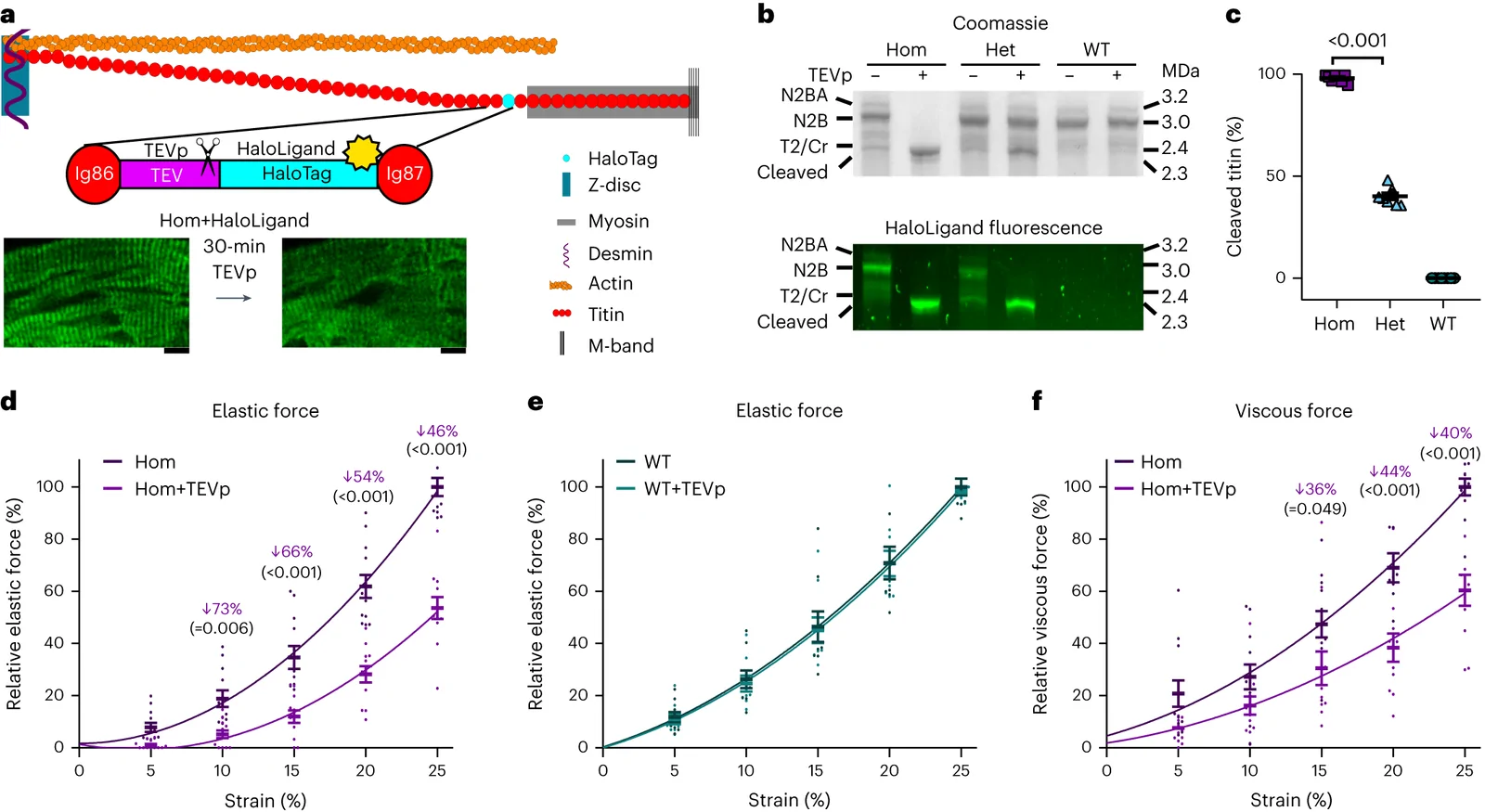
Specific titin cleavage in the TC-Halo mouse model and the contribution of titin to passive forces in cardiac LV fiber bundles. **a**, Schematic of the HaloTag-TEVp recognition cassette within titin in the TC-Halo mouse model. Confocal images show HaloTag labeling with Alexa Fluor 488-conjugated HaloLigand before and after titin cleavage on the same cardiomyocyte sample. Scale bar, 10 µm. Similar findings were obtained from *N* = 3 mice per group from *n* = 10 images. **b**, Coomassie stain of a loose titin protein gel showing the effects of TEVp treatment on cardiac titin in homozygous (Hom), heterozygous (Het) and WT LV tissue samples and the same gel detecting the HaloTag labeling with HaloLigand-Alexa Fluor 488. Samples were obtained from *N* = 4 mice for each genotype, and *n* = 8 is the number of lanes analyzed for each group. **c**, Quantification of cardiac titin cleavage on Coomassie-stained gels (*n* = 8). **d**, Elastic forces before and after 10-min TEVp treatment in Hom (*N* = 8, *n* = 12) cardiac fiber bundles. **e**, Elastic forces in WT fiber bundles (*N* = 6, *n* = 9) under the same treatment conditions. **f**, Viscous force changes in Hom fibers under the same treatment conditions. Forces relative to an initial 30% strain before TEVp incubation with the mean of the 25% strain set to 100%. Data are mean ± s.e.m. *N* refers to the number of animals used, and *n* refers to the number of technical replicates on a gel (**c**) or individual cardiac fiber bundles measured (**d**–**f**). Curves were fitted with a second-order polynomial. The significant force reduction after TEVp versus Hom control for a given strain is stated (purple values). Significance was determined using an unpaired *t*-test (**c**) or two-way repeated-measures ANOVA followed by Sidak’s multiple comparisons test (**d**–**f**). *P v*alues are in black. N2BA, N2B and Cr (Cronos) are titin isoforms, and T2 is a proteolytic titin fragment. ‘Cleaved’, A-band titin part after TEVp. Source data

Titin is a major mechanical protein in striated muscles, making up almost a fifth of the total protein content within the cardiomyocyte^14^. However, specific pharmacological targeting of titin to accurately determine its contribution to passive forces has not been previously possible. Our TC-Halo mouse contains a HaloTag-Tobacco Etch Virus protease (TEVp) recognition cassette cloned in frame between constitutively expressed *TTN* exons 225 and 226, encoding Ig domains I86 and I87 at the distal end of I-band titin (Fig. 4a). This cassette enables the specific, complete and acute cleavage of titin within the I-band using TEVp on homozygous mutant samples^15,16^. In addition, specific labeling of the HaloTag as part of the cassette can be used to track titin cleavage and ultrastructural changes in the sarcomere (Fig. 4a).

**Extended Data Fig. 3:**
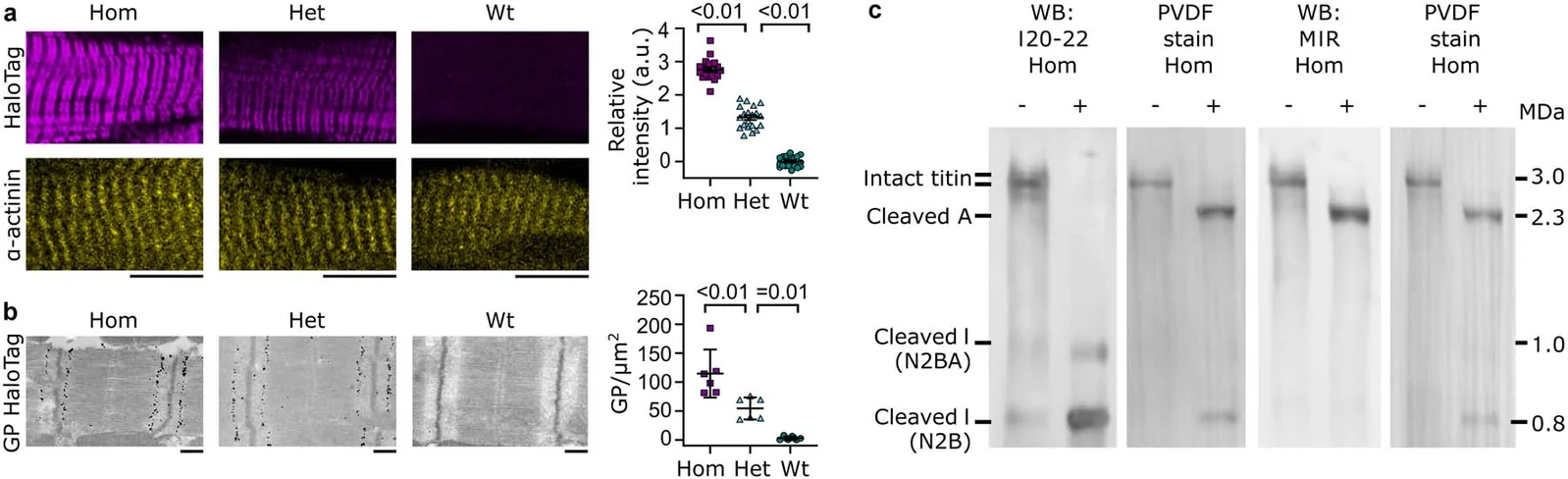
Confirmation of the TC-Halo cassette distribution and complete severing of titin with TEVp. (a) Confocal images of HaloTag labeling within mutant titin using anti-HaloTag and anti-α-actinin (secondary antibodies were Alexa Cy3-conjugated IgG and Alexa488-conjugated IgG, respectively), with associated quantification (right; mean ± SEM, n = 20), in homozygous (Hom), heterozygous (Het) and wildtype (Wt) TC-Halo mice. Scale bars, 10 µm. (b) Immunoelectron micrographs of anti-HaloTag (secondary antibody was nanogold-conjugated IgG) in Hom, Het and Wt TC-Halo mice, with associated quantification (right; mean ± SEM, n = 6). Scale bars, 500 nm. (c) Detection of the severed titin I-band fragment(s) with the I20-22 antibody and the A-band fragment with the MIR antibody, and accompanying PVDF stains (indicating protein load). Hom TC-Halo heart was used. Samples from titin gels came from N = 3 mice and n = 6 samples were run showing similar results. Significance was determined using a 1-way ANOVA followed by Tukey’s multiple comparisons test. n is the number of images analyzed. Source data

Because the TC-Halo mouse model has not been used previously in mechanical measurements of cardiac tissue, we initially performed experiments to ensure that the model is suitable to completely sever titin in our passive force recordings on cardiac fiber bundles. We confirmed Mendelian distribution of the cassette in LV cardiac tissue using western blot, immunofluorescence and electron microscopy (EM), with all titin protein containing the cassette in homozygous (Hom), 50% in heterozygous (Het) and none in wild-type (WT) fibers (Fig. 4b,c and Extended Data Fig. 3a,b). We could sever 100% of titin containing the cassette using 8 mg ml^−1^ TEVp for 10 min on Hom LV tissue, which we detected on protein gels using Alexa Fluor488-conjugated HaloLigand bound to the HaloTag (Fig. 4b), and we also identified the cleaved titin A-band and I-band segments by immunoblot using I20-22 (TTN-5) and MIR anti-titin antibodies, respectively (Extended Data Fig. 3c).

### Titin cleavage in fibers causes a reduction in passive force

**Extended Data Fig. 4:**
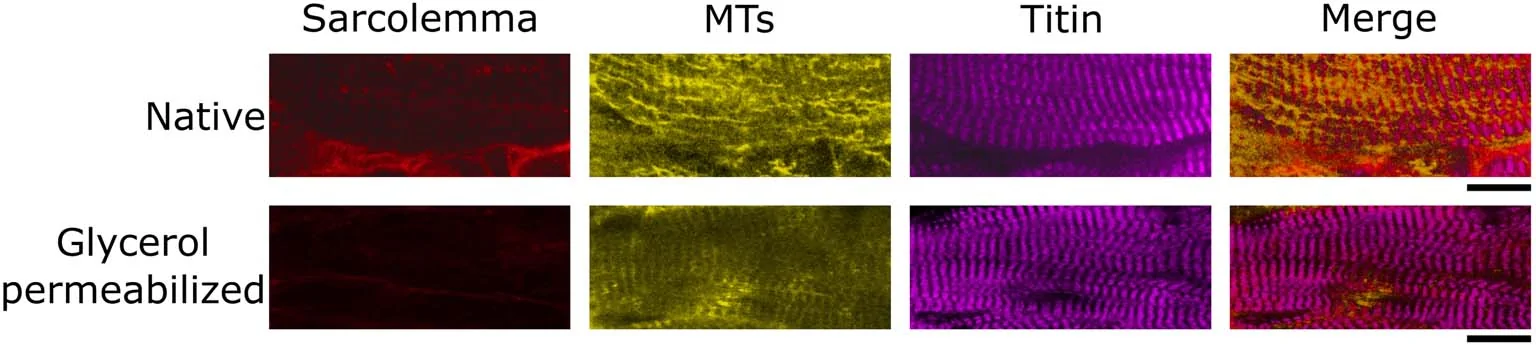
Cold storage of cardiac left ventricular (LV) fiber bundles in glycerol removes the sarcolemma and microtubule network. LV fiber bundles were pre-permeabilized by storage in a 50:50 rigor:glycerol solution at -20 °C for a minimum of 3 months. Sarcolemma stained with wheat germ agglutinin (WGA-555), microtubules (MTs) with anti-α-tubulin and titin with anti-HaloTag; secondary antibodies were Alexa488-conjugated IgG and Alexa 647-conjugated IgG, respectively. Scale bars, 10 µm. Similar findings were obtained from N = 3 mice per group from n = 30 images. Source data

**Extended Data Fig. 5:**
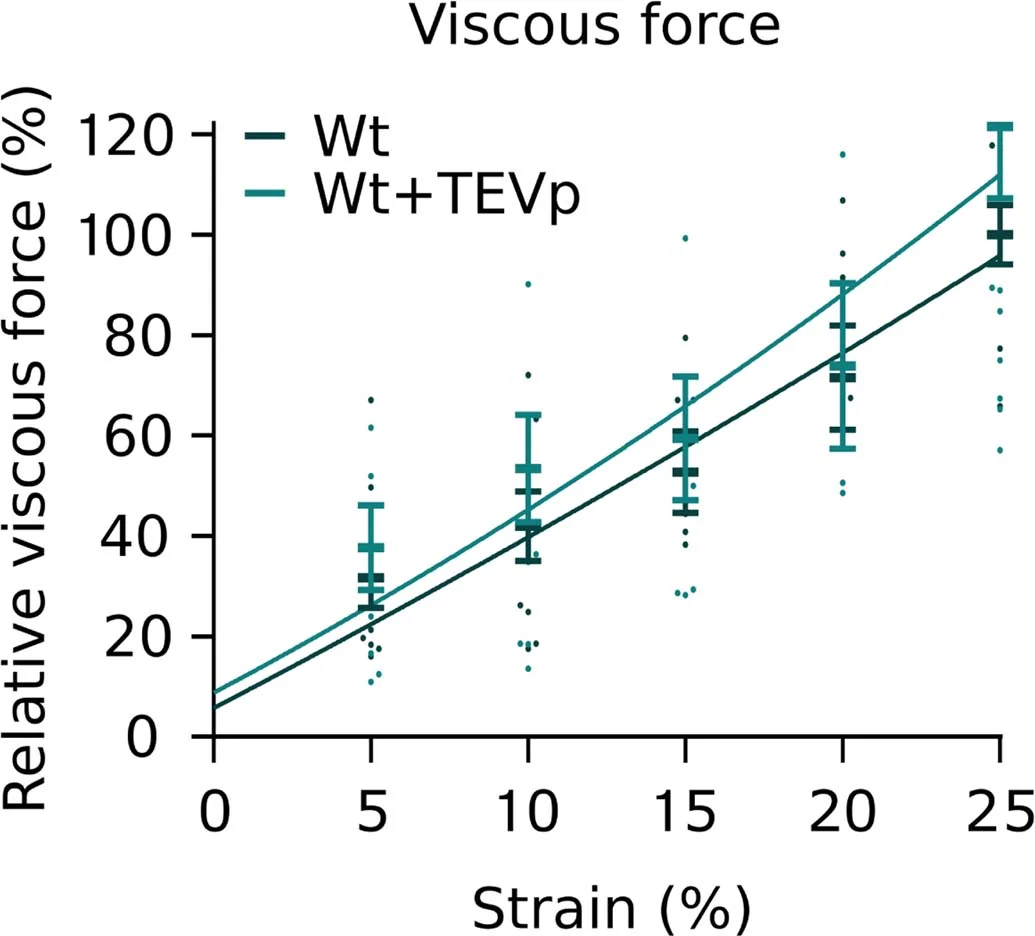
Viscous passive forces in Wildtype (Wt) cardiac left ventricular (LV) fiber bundles. Viscous forces before and after 10-min TEVp treatment in Wt (N = 6, n = 9) cardiac LV fiber bundles. Forces relative to an initial 30% strain before TEVp incubation with the mean of the 25% strain set to 100%. Data are mean ± SEM. N is the number of animals used and n is the number of cardiac fiber bundles measured. Curves fitted with a 2nd order polynomial. Source data

We proceeded to perform a stepwise passive stretch protocol on pre-permeabilized cardiac LV fiber bundles in which the sarcolemma and the MTs are already severely disrupted (Extended Data Fig. 4). We compared the passive forces before and after titin cleavage in homozygous (Hom) fiber bundles and used WT LV fiber bundles as our experimental control. Titin cleavage in the Hom fiber bundles caused a substantial decrease in elastic force at all strains ≥10%, ranging from a 73% decrease at 10% strain to a 46% decrease at 25% strain (Fig. 4d), which was much larger than what we had detected for either the MT or actin networks (compare Fig. 1e and Fig. 3b). TEVp treatment had no effect on passive elastic force in WT fiber bundles (Fig. 4e). Unlike in WT fiber bundles (Extended Data Fig. 5), viscous forces in Hom fiber bundles were also significantly reduced after TEVp incubation at fiber strains >10% (Fig. 4f).

### Passive force greatly drops in titin-cleaved cardiomyocytes

**Fig. 5:**
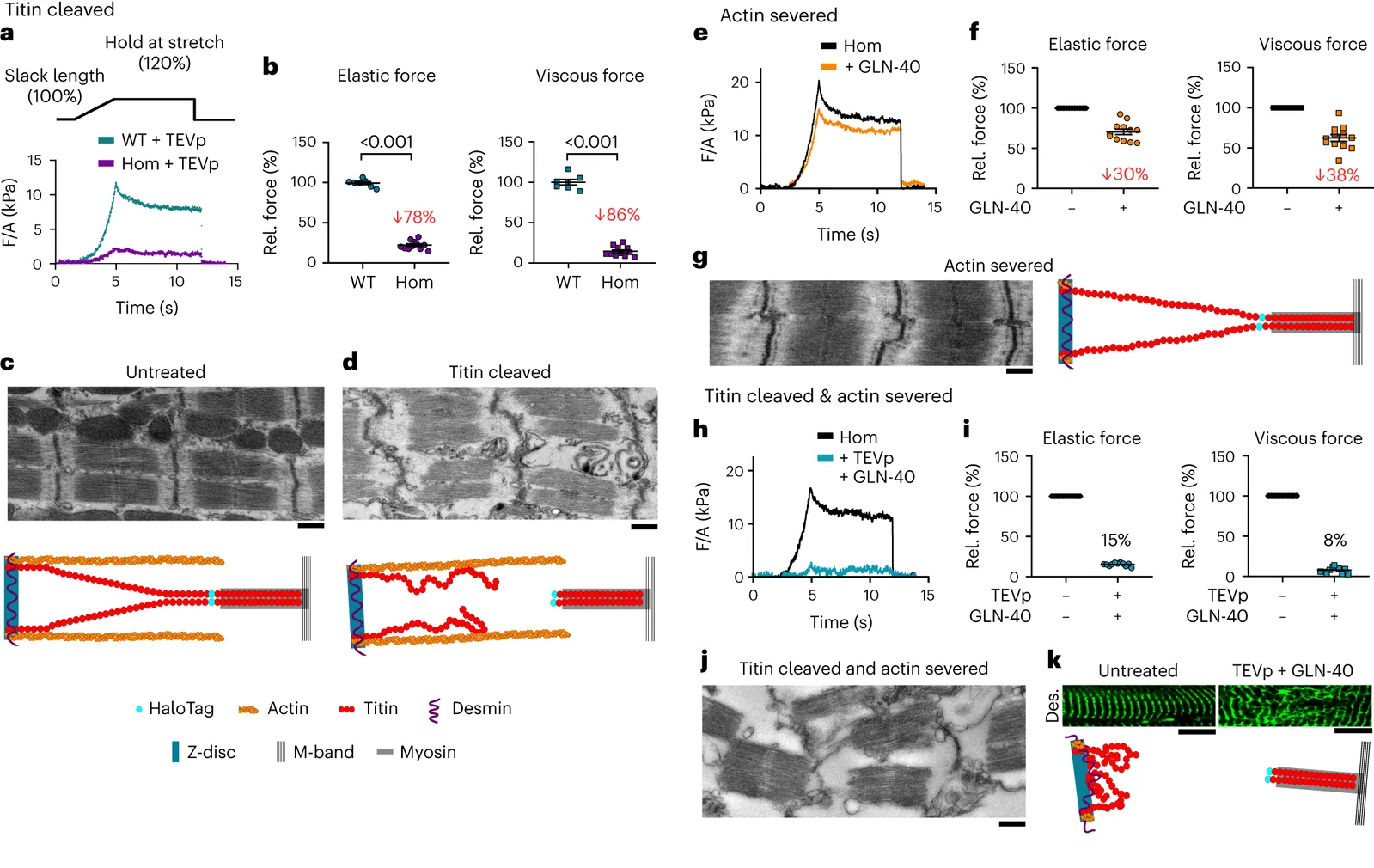
Passive force of single cardiomyocytes and sarcomere ultrastructure before/after titin cleavage and/or actin severing. **a**, Stretch protocol and raw force traces (force (F) normalized to cross-sectional area (A)) from Hom and WT permeabilized cardiomyocytes after TEVp treatment. **b**, Elastic and viscous forces at a 20% strain measured in permeabilized Hom (*N* = 5, *n* = 11) and WT (*N* = 4, *n* = 7) cardiomyocytes after TEVp incubation (relative to pre-TEVp treatment). **c**,**d**, Representative electron micrographs of Hom TC-Halo sarcomeres and cartoons of untreated Hom (half-) sarcomere (**c**) and Hom (half-) sarcomere after titin cleavage with TEVp (**d**). Raw force/cross-sectional area (F/A) traces from a Hom cardiomyocyte (**e**) and mean relative elastic and viscous forces (*N* = 5, *n* = 12) before and after actin severing with gelsolin (GLN-40) (**f**). **g**, Representative electron micrograph of Hom sample after actin severing with GLN-40 (left) and cartoon of half-sarcomere depicting the GLN-40 treatment effect (right). **h**, Raw force/cross-sectional area (F/A) of a Hom cardiomyocyte before/after both actin severing and titin cleavage. **i**, Elastic and viscous forces remaining after actin severing and titin cleavage (probably desmin based, *N* = 5, *n* = 11). **j**, Representative electron micrograph of Hom sarcomeres after titin cleavage and actin severing. **k**, Desmin immunofluorescence staining (secondary antibody, Alexa Fluor 488-conjugated IgG) of an untreated sample and after titin cleavage+actin severing, with representative cartoon depicting the treatment effect. Scale bars, 10 µm. Data are mean ± s.e.m. *N* refers to the number of animals used, and *n* refers to the number of individual cardiomyocytes measured. For electron micrographs, similar images were obtained from *N* = 3–5 mice per group from *n* = 35 images. Significance was determined using two-tailed unpaired *t*-test. Scale bars for all electron micrographs, 500 nm. Source data

We speculated that the smaller drop in titin-based passive forces observed at low versus high strain in cardiac fiber bundles could be due to the ECM (still present in the fiber bundle preparations) becoming increasingly important as a stiffness contributor with higher strain. Therefore, we also cleaved titin in single, isolated, permeabilized, TC-Halo cardiomyocytes isolated from LV tissue which, similarly to the pre-permeabilized fiber bundles, also lacked the sarcolemma and MTs but where the ECM is completely absent. Both the elastic and the viscous force contributions were measured in permeabilized cardiomyocytes strained to 20% (range of slack sarcomere length, 1.8–1.9 µm) before and after a 10-min TEVp treatment (Fig. 5a).

We found that, on average, the elastic force decreased by 78% in Hom cardiomyocytes treated with TEVp (Fig. 5b), much more than the 54% decrease observed at 20% strain in Hom cardiac LV fiber bundles (compare to Fig. 4d). Average viscous force decreased by 86% with titin cleavage in Hom cardiomyocytes (Fig. 5b), twice that detected in Hom cardiac LV fiber bundles strained to the same level (Fig. 4f). TEVp-treated WT cardiomyocytes did not show a noteworthy passive force drop (Fig. 5b).

Such large changes in passive force will also likely have a structural impact. Using transmission electron microscopy of untreated or TEVp-treated Hom cardiac LV tissue, we found that the cleavage of titin resulted in both Z-disc and A-band disorders (Fig. 5c,d).

### One-third of cardiomyocyte viscoelastic force is actin based

Next, we wanted to know if the absence of the ECM in single cardiomyocytes also affected the relative actin contribution to passive forces. Repeating the single cardiomyocyte stretch protocol, we again severed actin using GLN-40 (Fig. 5e). Mean elastic force decreased by 30% at 20% strain, which was very similar to what was detected in permeabilized LV fiber bundles (26% at 20% strain; compare to Fig. 3b). Likewise, actin-based viscous forces reached 38% of total viscous forces in permeabilized single cardiomyocytes (Fig. 5d), whereas a 24% contribution to viscous force had been detected in permeabilized LV fiber bundles at 20% strain (compare to Fig. 3c). Ultrastructurally, the severing of actin caused the A/I-band junction of the sarcomeres to become less well defined and the I-band region to appear lighter (Fig. 5g).

### Minimal passive force remains after actin and titin severing

**Extended Data Fig. 6:**
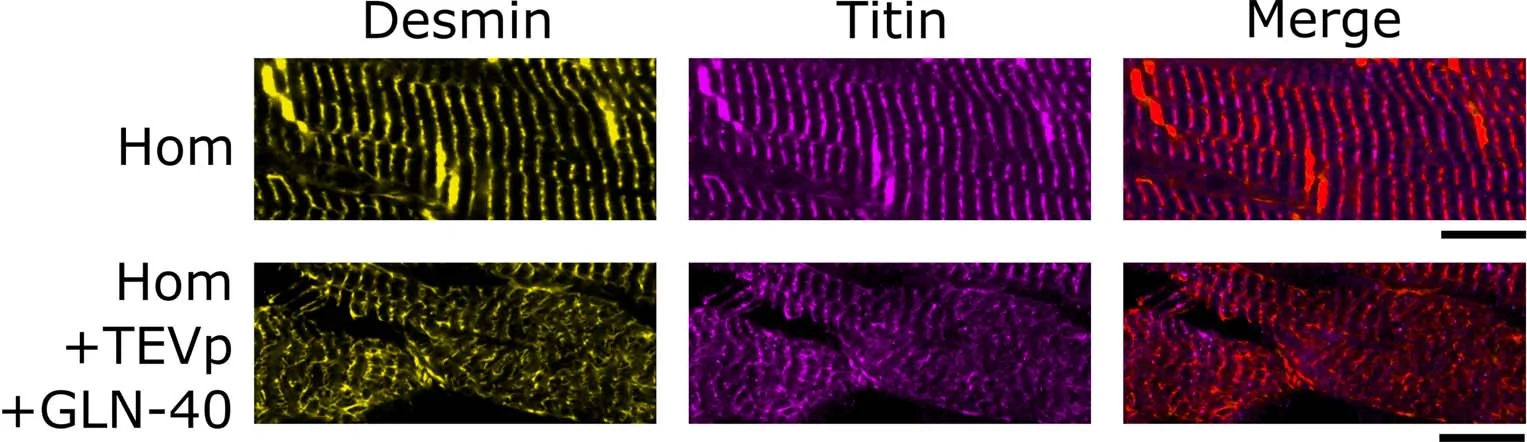
Desmin and titin staining after titin cleavage and actin severing. Confocal images of glycerol-stored homozygous (Hom) Titin-Cleavage-Halomouse left ventricular fiber bundles untreated (top) and treated with TEVp to cleave titin and gelsolin (GLN-40) to sever actin (bottom). Anti-desmin and anti-TTN-Z antibodies used; secondary antibodies were Alexa488-conjugated IgG and Alexa Cy3-conjugated IgG, respectively. Scale bars, 10 µm. Similar findings were obtained from N = 4 mice per group from n = 40 images. Source data

Judged by the effects on passive forces caused by titin cleavage (Fig. 5b) and actin severing (Fig. 5f) in the cardiomyocytes, we expected that both interruptions together in the same sample should result in the complete loss of passive forces. However, even after performing both of these treatments, a small amount of passive force remained (Fig. 5h): a residual 15% elastic force and 8% viscous force was detected (Fig. 5i). As we had not disrupted the IF/desmin network, we speculated that the remaining force could originate in the desmin filaments. Electron micrographs of LV tissue showed that, under these conditions, the sarcomeric A-bands and Z-discs were almost completely dissociated from each other, but the Z-discs still maintained a loose connection (Fig. 5j). Performing anti-desmin immunofluorescence staining, we found that, after both titin cleavage and actin severing, the desmin network became severely disordered but was still present as an interconnecting mesh (Fig. 5k and Extended Data Fig. 6).

### Titin and actin demonstrate non-reciprocal tensegrity

The ‘musical chairs’ approach of disrupting structural elements one by one showed treatment history-dependent effects on passive forces, as seen by the force variations detected by MT–sarcolemma interactions (compare to Fig. 2b,e) and ECM–titin/actin (compare to Figs. 3–5) interactions. However, in all these cases, the reciprocal interaction could not be studied by reversing the order in which they were disrupted. Titin and actin are also interaction partners within the sarcomere. More specifically, a segment of titin at the periphery of the Z-disc encoded by *TTN* exon 28 is anchored to sarcomeric actin, shortening the effective contour length of elastic I-band titin^12^. Whether titin also influences the relative passive force contribution of actin is not clear.

**Fig. 6:**
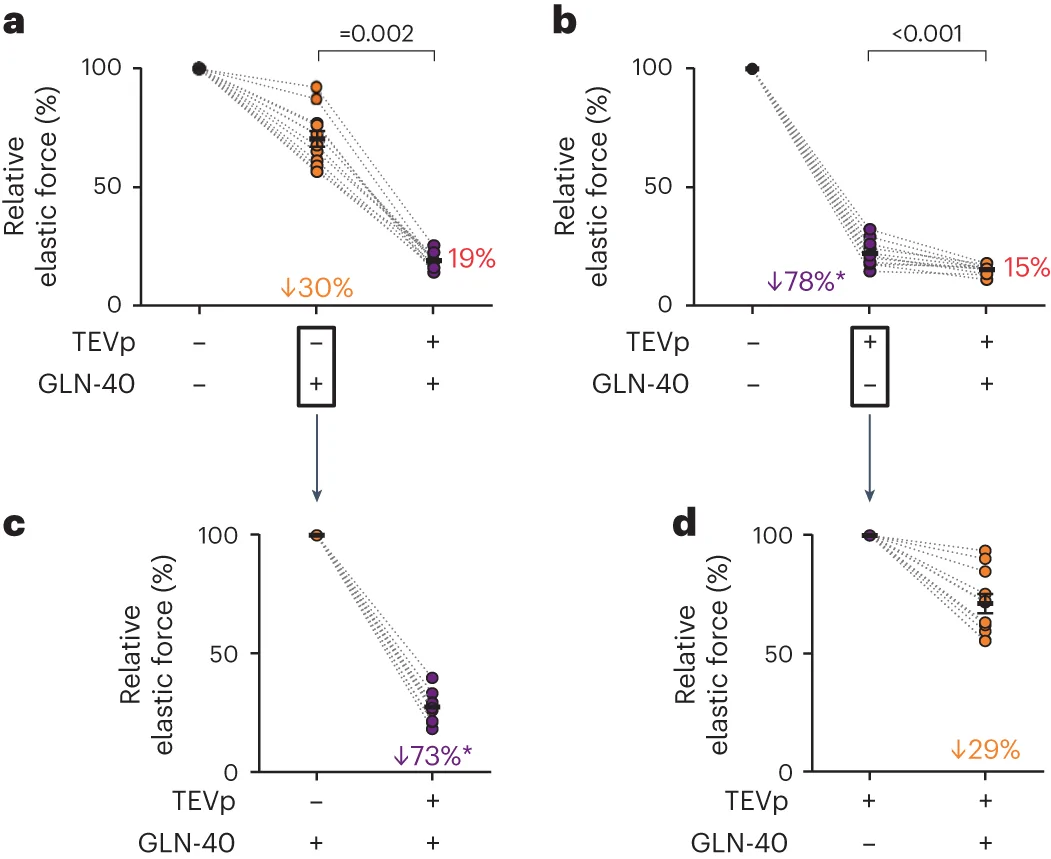
Actin and titin interdependence for elastic passive force contributions in cardiomyocytes. **a**–**d**, Elastic forces after first actin severing and then titin cleavage (*N* = 5, *n* = 12) (**a**) or first titin cleavage and then actin severing (*N* = 5, *n* = 11) (**b**) in permeabilized Hom cardiomyocytes. Titin cleavage relative to the previous actin severing (**c**) and actin severing relative to the previous titin cleavage (**d**) (as seen in **a** and **b**, respectively). Data are expressed as mean ± s.e.m. *N* refers to the number of animals used, and *n* refers to the number of individual cardiomyocytes measured. Orange, purple and red values are comparable actin, titin and IFs/desmin contributions, respectively. Dotted lines indicate the same sample measured after consecutive treatments. Comparisons and significance were determined using a two-tailed paired *t*-test. * indicates a significant difference. Further statistics can be found in Supplementary Table 1. Source data

**Extended Data Fig. 7:**
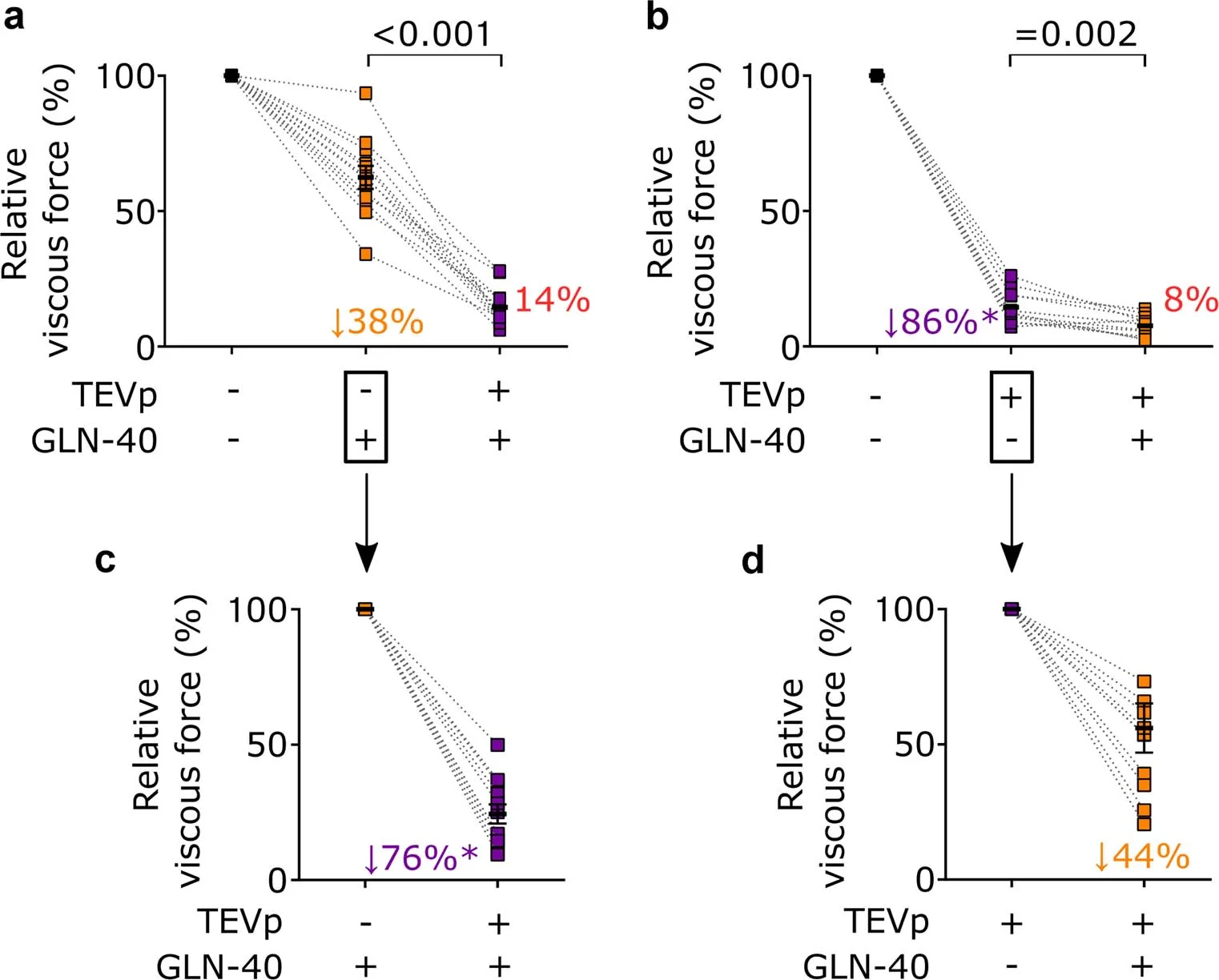
Actin and titin interdependence for passive viscous force contributions in cardiomyocytes. (a) Viscous forces after first actin severing and then titin cleavage (N = 5, n = 12) or (b) first titin cleavage then actin severing (N = 5, n = 11) in permeabilized Hom cardiomyocytes. (c) Titin cleavage relative to the previous actin severing and (d) Actin severing relative to the previous titin cleavage (as seen in a and b, respectively). Data expressed as mean ± SEM. N is the number of animals and n is the number of cardiomyocytes measured. + symbols highlight the treatment of interest at each step. Orange, purple and red values are comparable actin, titin and IFs/desmin contributions, respectively. Comparisons and significance were determined using a 2-tailed Student’s *t*-test. *indicates a significant difference. Source data

We addressed this issue by disrupting both actin (by GLN-40) and titin (by TEVp) in single permeabilized LV cardiomyocytes but varied the order in which they were disrupted (Fig. 6a,b and Extended Data Fig. 7). Focusing on the elastic force, regardless of which treatment occurred first, no noteworthy difference was detected in the remaining elastic force after both titin cleavage and actin severing (Fig. 6a,b and Supplementary Table 1). First titin cleavage and then actin severing resulted in a mean 15% passive force remaining, whereas first actin severing and then titin cleavage resulted in 19% passive force remaining, consistent with the IF network being unaffected by the order in which titin and actin were disrupted (Fig. 6a,b and Supplementary Table 1).

Actin severing caused a consistent loss of ~30% passive elastic force, regardless of whether titin had previously been severed or not (Fig. 6a,d, orange values, and Supplementary Table 1). This indicated that the contribution of actin to passive stiffness was not influenced by the presence of titin. However, the importance of actin in aiding the tensional integrity of titin became apparent in the reciprocal experiment. Whereas titin cleavage alone resulted in a 78% decrease in elastic force in Hom sarcomeres (Figs. 5b and 6b), elastic force in actin-severed Hom cardiomyocytes dropped by 73% upon titin cleavage (Fig. 6c), which was significantly less than if actin was present (Supplementary Table 1). Similar results were seen in the viscous forces (Extended Data Fig. 7 and Supplementary Table 1). These results are consistent with actin and titin filaments being part of a cellular tensegrity structure.

### Titin with MTs, actin and ECM governs myocardial stiffness

**Fig. 7:**
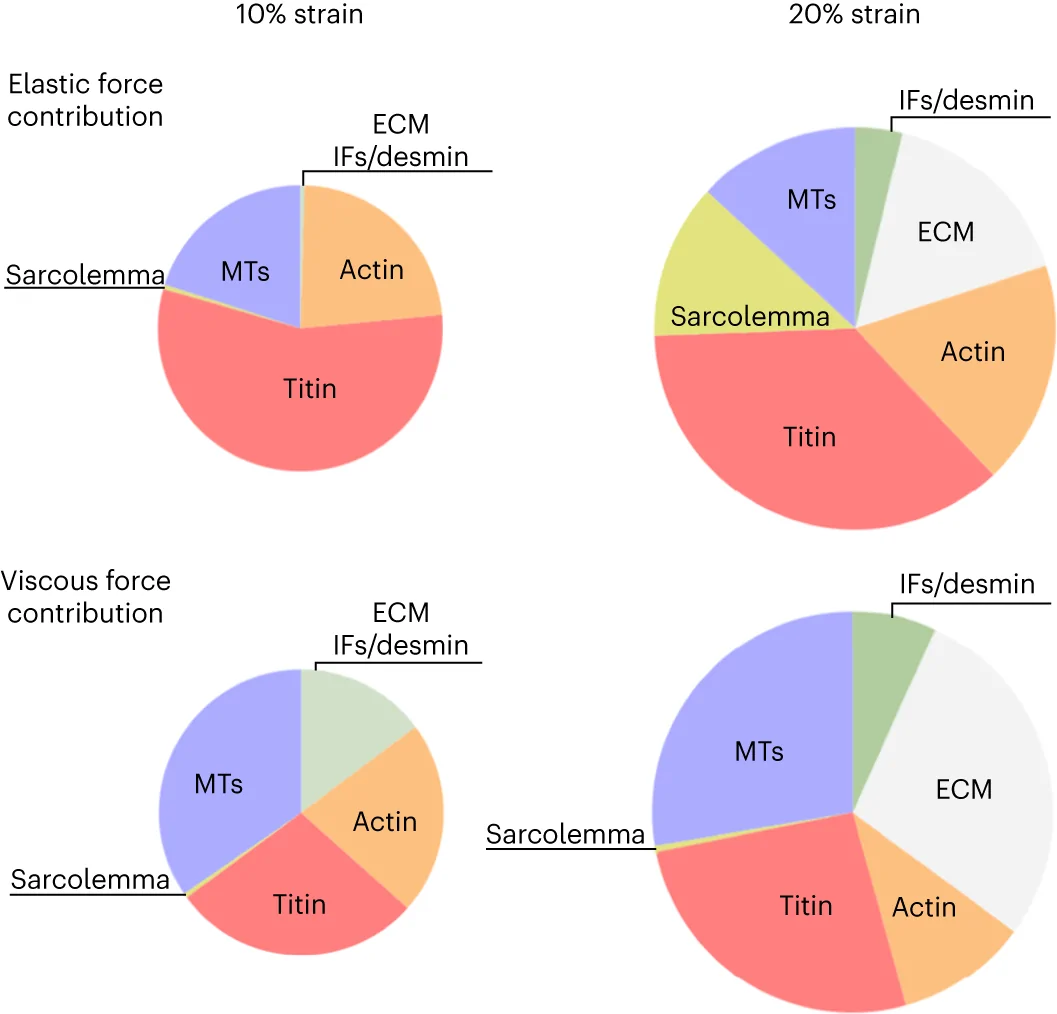
Relative passive force contributions of the major myocardial structural elements. Elastic (top) and viscous (bottom) force contributions of titin, MTs, sarcolemma, ECM, actin and IFs/desmin at low (left) and high (right) strain. Values can be found in Supplementary Table 2. Source data

Based on our series of experiments and maintaining our ‘musical chairs’ approach, taking into account the lack of sarcolemma and MTs in the pre-permeabilized LV fiber bundles and cardiomyocytes, and with the inclusion of the sarcolemma data after MT network disruption, we could summarize our findings as seen in Fig. 7. The distribution of the total passive force among the structural elements varies greatly depending on the strain level and on whether elastic or viscous forces are considered (Fig. 7 and Supplementary Table 2). Titin alone contributes over one-half of the elastic forces at a 10% strain amplitude and over one-third at 20% strain. Titin also contributes more than one-quarter of the viscous forces at both strain levels. The MTs and actin substantially contribute to the elastic and viscous forces as well, each contributing over 20% at low strain. The MTs are the predominant contributor to viscous forces (nearly 35%) at low strain. Whereas the ECM, sarcolemma and desmin filaments have a negligible contribution to the elastic forces at low strain, the ECM and desmin jointly contribute ~15% to viscous forces. However, the pattern changes with high strain: the ECM, actin, MTs and the sarcolemma each contribute close to one-eighth or more to the elastic forces, for a total of ~60%, the remainder (next to the dominating titin) taken up by the IFs/desmin. The viscous forces at high strain are nearly equally shared among the ECM, MTs and titin, whereas actin still contributes ~12% and desmin ~7%. Collectively, these results demonstrate that titin is the dominant contributor to elastic forces in LV myocardium, with major support from actin, the MTs and (only at larger strain) the ECM and sarcolemma. Viscous forces are more uniformly distributed among the MTs, titin and actin structures, with the ECM becoming increasingly important at high strain.

## Discussion

Our systematic ‘musical chairs’ approach to investigate the various sources of myocardial passive stiffness in a single mouse model highlighted the critical elements of the myocardial architecture in determining this stiffness, which vary in their relative contribution to total stiffness depending on strain level and if elastic and viscous forces are considered separately. The elastic forces are governed by titin in concert with the MTs, actin, the ECM and the sarcolemma, whereas the viscous forces derive—in nearly equal shares—from the MTs, titin and the ECM, with substantial support from the actin cytoskeleton. Moreover, although together these elements determine myocardial passive stiffness, they do not equally support each other’s contributions, as demonstrated by the non-reciprocal interactions. These findings highlight that no structural element truly acts in isolation, and consideration for tensegral interactions must be given when developing therapeutic strategies targeting myocardial passive stiffness.

Increases in titin stiffness have been associated with HF and increased myocardial stiffness^2^. A previous study by Granzier and Irving^19^, which also used a systematic elimination approach to evaluate the important elements in myocardial stiffness, estimated that titin contributed up to 70% of passive tension when the protein was crudely extracted from permeabilized fiber bundles using KCl/KI^19^. The use of the TC-Halo mouse enabled the precise cleavage of titin, and we showed as much as a 73% decrease in elastic force (in permeabilized samples) at low strain levels (Fig. 4d), consistent with the previous report^19^. Our work suggests that the modulation of titin stiffness therapeutically would have a large impact on overall myocardial stiffness and supports the ongoing pursuit of finding a therapeutic that targets titin stiffness.

Another major contributor to myocardial stiffness is the MT network. At 10% strain, the MTs contributed more to the total viscous force than any other structural element (Fig. 7 and Supplementary Table 2). Our findings align with the current understanding of the importance of the MT network in modulating cardiac stiffness^18,20,21^. The MT network has been shown to contribute to tensile forces in both a rate-dependent and length-dependent manner^18^. Increased detyrosination of the MT network has been found in failing human cardiomyocytes and is associated with increased viscoelasticity and impaired contractility^20^. In addition, in HF fiber bundles, baseline elastic stiffness and viscosity were found to be higher than in healthy controls^22,23^. Colchicine treatment to disrupt the MT network was successfully used to reduce viscous forces^22,24^. Although colchicine has been tested therapeutically in cardiovascular disease, little is known about how this treatment modifies overall myocardial passive stiffness^25^. Based on our study, a cardiac-specific therapeutic to target the MT network-derived viscous forces could be advantageous for reducing total passive stiffness.

As expected from previous work^26^, the ECM also had a larger contribution to passive forces at high strain but has a low passive stiffness at low strain (Fig. 7). The shift in dominant force source with higher strain levels was apparent when the actin and the titin force contributions became relatively reduced in fiber bundle samples at high strain (Figs. 3–5). Due to the complex viscoelastic nature of the ECM^27^, we cannot rule out that some damage may have occurred through permeabilization or through mechanical stretching alone, causing us to underestimate the contribution of the ECM^28^. However, the agreement between our dataset and published work suggests that potential damage to the ECM causing experimental artifacts was unlikely.

Sarcolemma remodeling and sarcolemma repair dysfunction are additional features of HF, but the direct mechanical consequence of these changes has not been measured^29^. A range of estimates has been suggested for the contribution of the sarcolemma to passive forces under healthy conditions^19,30,31^. In this investigation, we were particularly careful to ensure that forces measured were not due to solution change artifacts (Extended Data Fig. 2) and to monitor the state of the MT network (Figs. 1a,b and 2a). We found that the sarcolemma contributes somewhat to elastic force at higher strain (Fig. 2b). However, the measured elastic force contribution of the sarcolemma was treatment history dependent, with the state of the MT network influencing the elastic force measured (Fig. 2b–d). Regardless, our results provide a key link between native and permeabilized tissue research^9^.

Actin provides crucial cellular scaffolding and anchoring points for many other proteins and is integral for active force production^32^. Our study shows that the contribution of actin to passive forces is relatively independent of the other cytoskeletal elements and the strain level (Fig. 7). We observed a consistently relevant elastic force contribution from actin in both LV fiber bundles and LV cardiomyocytes and in both the presence and absence of titin (Figs. 3b, 5f and 6a,d). We also demonstrated the importance of actin in maintaining titin tension, with the removal of actin resulting in a loss of titin-based forces by 5–10% (Fig. 6b,c and Extended Data Fig. 7b,c). Although our approach targets all actin species, a previous study showed that actin severing by GLN-40 in single isolated rat cardiac myofibrils (where only sarcomeric actin is present) reduced stiffness by approximately 57% (ref. ^12^). This is similar to the contribution of actin to viscous forces at low strain found in fiber bundles (Fig. 3c), suggesting that sarcomeric actin, much more than extrasarcomeric actin, determines the viscous forces of rodent cardiomyocytes. In contrast, the non-sarcomeric actin cytoskeleton is highly relevant for mechanical stiffness in other cell types^33^. Therefore, specifically targeting either non-sarcomeric or sarcomeric actin may reveal a more complex breakdown of actin passive force contributions; however, this was not within the scope of this study.

Despite trying to eliminate all sources of passive force, a small amount remained, which we attribute to the IFs/desmin. The IFs are considered highly extensible^9^ and, therefore, thought unlikely to play a major role in cardiomyocyte stiffness. However, when everything else was stripped away, desmin still provided a loose association between the sarcomeric elements and probably produced some residual force (Figs. 5j,k and 6a,b and Extended Data Fig. 7a,b). Granzier and Irving also suggested that the IFs/desmin may contribute to the remaining passive forces, although they found higher contributions (~10%) of the IFs/desmin at lower strain compared to higher strain^19^. As we found no residual force after titin cleavage in LV fiber bundles at low strain (Fig. 4d), the residual force seen by Granzier and Irving at low strain may have been a consequence of the non-specific titin extraction used rather than the contribution of the IFs/desmin themselves. It was recently shown that desmin stabilizes the MT network at the Z-discs^34^, suggesting that, although desmin does not directly contribute greatly to myocardial stiffness, it may play a critical role in determining the stiffness profiles of other protein networks.

Limitations arise from performing a series of experiments at different stages of our ‘musical chairs’ approach rather than conducting all disruptions in a single LV fiber bundle. Although this would be an ideal experiment, problems can arise from maintaining a fiber bundle for an extended period under harsh conditions, making the results less reliable. In addition, we did not cleave titin and sever actin in native/intact preparations as neither TEVp nor GLN-40 can penetrate the sarcolemma. We also only inferred the contribution of the ECM and IFs/desmin. Although our study provides a good foundation for understanding the complex interplay among myocardial mechanical network members, the myocardium is very heterogeneous. It can vary in its composition, fiber orientation and mechanical properties depending on the location within the ventricle or layer within the myocardial wall itself^35–37^ or species^38–40^. Therefore, caution is required when extrapolating these findings beyond LV tissue and into human myocardium.

Finally, the interplay among the different structural networks that contribute to myocardial passive stiffness highlights the need to start considering how these interactions may influence overall cellular stiffness and integrity^11^. These types of interactions may explain, in part, the failing of some therapeutic strategies tried in the past^41,42^. However, with the improvement of our understanding of the sources of myocardial stiffness and how interactions between structural networks affect individual element stiffness profiles, these interactions may be artfully exploited for therapeutic gain in the future.

## Methods

### Animal model and genotyping

The mouse experiments adhered to the standards prescribed by the University Hospital of Muenster. Authorization for the breeding and experimental use of the TC-Halo mice^15^ was provided by the animal welfare board of the state of North Rhine-Westfalia (Landesamt für Natur, Umwelt und Verbraucherschutz Nordrhein-Westfalen, LANUV NRW, 81–02.04.2019.A472). *Mus musculus* of background strain C57BL/6JRj were used, age 8–16 weeks, both male and female. Mice were housed in open cages closed with a mesh lid. Mouse housing was subjected to a 12-h day/night cycle. Temperatures of 20–23 °C and humidity of 30–60% were monitored and maintained. Water and food was accessible ad libitum.

For genotyping, we employed polymerase chain reaction (PCR). Samples from ear punches were incubated overnight at 56 °C and shaken at 650 r.p.m. in a proteinase K buffer (100 mM Tris-HCl, pH 8, 5 mM EDTA, 0.2% SDS, 200 mM NaCl, 100 mg ml^−1^ proteinase K). After this, DNA was isolated using isopropanol precipitation and then rinsed with 70% ethanol. The purified DNA was subsequently resuspended in TE buffer (10 mM Tris-HCl, 1 mM EDTA). The primers selected for this method were 5′-cgtggtggcttatcttctagc-3′ and 5′-ctgttggttcatgcatctcc-3′.

### Preparation of mouse hearts

Adult TC mice (age 8–16 weeks) were killed by cervical dislocation, according to the recommendations of the local animal care and use committee of the University of Muenster. Hearts were excised, excess blood was removed and hearts were washed in warmed (37 °C) normal Tyrode’s (NT) solution (140 mM NaCl, 0.5 mM MgCl_2_, 0.33 mM NaH_2_PO_4_, 5 mM HEPES, 5.5 mM glucose, 5 mM KCl, adjusted to pH 7.4 with NaOH) containing no added Ca^2+^ that was continuously perfused with 100% O_2_, for native LV fiber bundle experiments. Alternatively, hearts were washed in cold PBS and then retrograde perfused through the aorta with 50:50 rigor:glycerol solution (75 mM KCl, 2 mM MgCl_2_, 2 mM ethylene glycol-bis(β-aminoethyl ether)-N,N,N′,N′-tetraacetic acid (EGTA), 10 mM Tris, 50% glycerol, pH 7.1) for at least 15 min, until they remained inflated and rigor had occurred, and then they were stored in 50:50 rigor:glycerol solution at −20 °C for a minimum of 4 weeks before being used as pre-permeabilized LV fiber bundles. To limit protein degradation, rigor:glycerol solution contained one tablet of protease inhibitor (cOmplete, Roche Diagnostics) per 100 ml of solution.

Some hearts were also retrograde perfused with fixative (4% paraformaldehyde, 15% picric acid in 100 mM PBS, pH 7.4) and then stored for at least 24 h before further processing for EM and immunofluorescence analysis. Several other hearts were also flash frozen in liquid nitrogen and stored at −80 °C for later use in biochemical assays.

Generally, care was taken to ensure that the mechanical proteins remained unaffected by the isolation process by controlling parameters such as temperature of isolation and solution compositions. Treatment success was checked by gel electrophoresis and western blot and on immunofluorescence images of specific structures compared before and directly after isolation and after different treatment times, as described below. When structures were clearly disrupted due to sample storage—that is, the loss of sarcolemma and MTs in glycerinated LV fiber bundles—this was taken into consideration in the final calculations in Fig. 7.

### TEVp expression

TEVp was either acquired commercially from Thermo Fisher Scientific (AcTEV protease, 12575-023) and used according to the manufacturer’s instructions or produced in-house from vector pMHT238Delta. In brief, in-house expression of TEVp was induced in BLR (DE3) cells at OD_600_ = 0.6–1.0, using 1 mM IPTG, for 3 h at 37 °C. TEVp was purified with Ni-NTA beads agarose (Qiagen, 30210) and eluted with 250 mM imidazole. The buffer was exchanged with relaxing solution (RS: 170 mM K-propionate, 20 mM MOPS, 2.5 mM Mg-acetate, 5 mM K_2_EGTA, 2.5 mM ATP, 14.5 mM creatine phosphate, 1× working concentration of protease inhibitor cocktail (Promega, G6521), pH 7.0, at 0 °C) using 10K Amicon ultra filters (Millipore, UFC201024). A yield of ~8 mg ml^−1^ was obtained. Purified TEVp was flash frozen in liquid nitrogen and stored at −80 °C. To test TEVp viability, cardiac tissue (10 mg) was permeabilized in RS supplemented with 0.5% Triton X-100 for 8 min and subsequently washed three times in RS. The cardiac tissue was then incubated with TEVp (7.5 µl) and 100 mM DTT for 6 h at 22 °C and prepared for SDS-PAGE. TEVp viability and quality were assured if the intact N2B titin band (~3.0 MDa) was reduced by ~100% in treated Hom tissue samples.

### SDS-PAGE and immunoblotting

Agarose-strengthened 1.8–2.4% SDS-PAGE titin gels or regular gels/western blots were prepared according to our published protocols^43,44^; sample preparation for the tubulin blots was modified from ref. ^45^. Samples were homogenized in modified Laemmli buffer (8 M urea, 2 M thiourea, 3% SDS, 0.03% Serva Blue, 50 mM Tris-HCl, pH 6.8, 10% glycerol, 75 mM DTT), stored on ice for 20 min and subsequently boiled for 3 min at 97 °C. The protein concentration was determined spectrophotometrically using Bradford reagent (Thermo Fisher Scientific).

Titin proteins were visualized by Coomassie staining or western blot as described previously^17^ or with covalently bound HaloLigand. HaloLigand labeling was performed before TEVp treatment. Tissue samples were incubated with 1:1,000 HaloLigand Alexa Fluor 488 (Promega) for 15 min at 37 °C and then washed three times with RS. Samples were then incubated with TEVp for 6 h before being prepared and run on titin gels and visualized using the ImageQuant LAS 4000 Imaging System with the Y515Di filter (Cy2).

Relative band intensities on Coomassie-stained gels measured for intact and cleaved N2BA/N2B titin were used to calculate the percentage of total titin cleaved by TEVp. To detect titin cleavage by western blot, we used a combination of the antibodies listed in Supplementary Table 3 and visualized them using the ImageQuant LAS 4000 Imaging System (GE Healthcare). Signals from HRP-conjugated secondary antibodies were visualized by chemiluminescence (Amersham ECL Prime start western blot detection reagent, GE Healthcare). Signal intensity for all bands detected was quantified using either MultiGauge version 3.0 (Fuji) or ImageQuant TL version 7.1 (GE Healthcare) software.

### Polymerized tubulin determination

Polymerized tubulin was separated from free, soluble, monomeric tubulin using a modified method described in ref. ^45^. In brief, native LV fiber bundles, with or without colchicine treatment, were finely chopped and placed in 300 µl of hypotonic solution (0.1 mM MgCl_2_, 2 mM EGTA, 1% tergitol, 2.5 mg ml^−1^ cOmplete protease inhibitor EDTA free, 50 mM Tris-HCl buffer, pH 6.8) and heated to 37 °C for 5 min in the dark to wash the free soluble monomeric tubulin from the sample. Samples were then centrifuged at 14,400*g* for 10 min at 25 °C. The supernatant was removed and the pellet resuspended in 300 µl of hypotonic buffer and kept as the polymerized fraction of the MTs. Samples were mixed with 60 µl of Laemmli buffer and treated, as stated above, before being run on a 10% SDS gel and western blot performed. After protein transfer, the PVDF membrane used was stained with Coomassie solution and imaged to visualize the total protein transferred from the gel before being incubated with the anti-α-tubulin. The PVDF total protein loading control image was compared to the anti-α-tubulin blot for quantification.

### Native LV fiber bundle mechanical measurements

Fresh (native) cardiac LV fiber bundles were isolated from the LV wall, ranging in size from 1–2.5 mm long and 0.1–0.4 mm thick. To determine the relative force versus strain relationships, the LV fiber bundles were secured at each end with suture before being excised from the heart and were attached lengthwise between a piezomotor and a force transducer via aluminum clamps (Scientific Instruments) in a temperature-controlled bath set to 37 °C and continuously perfused with oxygenated NT solution containing 1 mM EGTA and 30 mM 2,3-butanedione 2-monoxime to prevent active contraction. Force data were acquired using machine-customized software (https://github.com/DrDJIng/FiberStretchProgram), which, however, was not customized for these experiments specifically.

The LV fiber bundles were exposed to a strain of up to 20% to minimize damage to the sample and to ensure a secure hold on the preparation. Typically, a stretch protocol was performed with five steps, up to 20% strain; length changes were accomplished by manual or computer-driven means. For each step, the LV fiber bundle was stretched and held at a given strain for 10 s before being released back to 0% strain (slack length). There was a ~1-min waiting time between each step, and each step was repeated twice and analyzed as separate technical measurements (n), before continuing onto the next step. Force data were recorded at 1,000 Hz. Forces were then normalized to the highest elastic or viscous force obtained during the 20% strain before a given treatment. Elastic force was determined by fitting the force relaxation segment of each step of the raw traces with a one-phase exponential decay function in GraphPad Prism version 8 (GraphPad Software). Viscous force was determined as the difference between the peak force and the elastic force value for each step.

After the initial stretch protocol, 10 µM colchicine was added to the NT perfusion solution and left to incubate for 90 min before the stretch protocol was repeated. Perfusion was stopped for the permeabilization of the LV fiber bundle with the sodium-based RS (RS made with 170 mM Na-propionate in place of 170 mM K-propionate) containing 0.5% Triton-X 100. The LV fiber bundle was left to permeabilize for at least 30 min and then washed two times with standard RS before the stretch protocol was repeated. The process was then repeated with a 30-min incubation with 1 µg µl^−1^ GLN-40 to sever actin.

### Pre-permeabilized LV fiber bundle measurements

Pre-permeabilized LV fiber bundles were isolated from rigor:glyerol perfused hearts (dimensions similar to those of the native fibers), and ends were secured with suture and vigorously washed in RS on ice. LV fiber bundles were attached lengthwise between a piezo motor and a force transducer via aluminum clamps (Scientific Instruments). Measurements were performed at room temperature (20–22 °C). Force data were recorded at 1,000 Hz. Each LV fiber bundle was initially suspended in a bath of RS and then readily transferred to other baths as needed.

LV fiber bundles were measured at slack length (0% strain) and then strained by 30% in six incremental stretch–hold steps. In between each step, the LV fiber bundle was held isometrically for 10 s to record stress relaxation. LV fiber bundles were repeatedly moved through this protocol (rest time in-between trials, 10 min) until force traces became consistent between trials (2–3 times), and the final run was recorded. Then, the sample was treated with TEVp in RS for 10 min, and the stretch–hold protocol was repeated at regular intervals for 30 min. The final run was recorded, and forces were compared before and after treatment. To account for typical force decreases over multiple stretch–hold protocols, these experiments were repeated without TEVp incubation, and these data were used as a control baseline. Forces were then expressed relative to the mean elastic or viscous force at 25% strain before TEVp incubation.

### Force measurements on isolated permeabilized cardiomyocytes

Cardiomyocytes were obtained from hearts that were isolated and washed in PBS and then flash frozen in liquid nitrogen and stored at −80 °C until used. A small piece of tissue was cut from the LV of the frozen heart and thawed in RS at 0 °C, mechanically disrupted and then permeabilized in RS supplemented with 0.5% Triton X-100 for 8 min and subsequently washed three times in RS. Cardiomyocytes were mounted between a piezoelectric motor and a force transducer (Aurora Scientific, 403A) using shellac dissolved in 70% ethanol (120 mg ml^−1^) on the stage of an Axiovert 135 inverted microscope (Carl Zeiss). Cells were then stretched from slack length (0% strain, corresponding to a measured sarcomere length of 1.8–1.9 µm) to 20% strain and typically held for 7 s. Passive forces were recorded before and after a 10-min incubation with TEVp. For actin removal, the cardiomyocytes were incubated for 30 min with 1 µg µl^−1^ of the Ca^2+^-independent gelsolin fragment GLN-40, and then measurements were repeated. Elastic and viscous forces were determined as stated above for fiber bundles.

### Confocal microscopy sample preparation and imaging

Samples were first dehydrated through a graded ethanol series before being embedded in paraffin. Using an RM2235 Leica microtome, sections of 5–7-mm thickness were prepared. These sections underwent rehydration and were treated with peroxidase buffer, and then an antigen retrieval process using citrate-EGTA was applied. After washing with PBS, the slides were blocked using a solution of 5% BSA and 0.5% Triton X-100 for 1 h. They were then exposed to primary antibodies at 4 °C overnight, in conjunction with the antibodies detailed in Supplementary Table 3 (all mixed in PBS), which also included secondary antibodies. Once stained, the samples were set in Mowiol with an addition of n-propyl-gallate to prevent bleaching. Analysis was conducted, and immunofluorescence images were captured using a Leica SP8 confocal laser scanning microscope with an HC PL Apo CS2 ×63 NA 1.4 oil immersion lens. Images were recorded using NIS Elements version 4.3 software.

### Transmission and immuno-electron microscopy

Samples, once fixed, were sectioned longitudinally using a VT 1000S Leica vibratome and subsequently washed in PBS twice. For in situ visualization of the HaloTag, the samples underwent a blocking process with 20% normal goat serum (NGS) for 1 h. They were then treated with the HaloTag polyclonal anti-rabbit (Promega, at a 100-fold dilution) in a PBS solution enhanced with 2% NGS and left overnight at 4 °C. After this, the sections were thoroughly rinsed with PBS three times and exposed to the corresponding secondary antibody linked to 1.4 nm Nanogold (Nanoprobes) overnight at 4 °C with stirring. After comprehensive washing, the sections were post-fixed using 1% glutaraldehyde for a brief 10-min period. After rinsing, the HQ Silver kit (Nanoprobes) was applied to amplify the visible gold particle (GP) diameter. After OsO_4_ application, the samples received a counterstain of uranyl acetate in 70% ethanol, underwent dehydration and were then set in Durcupan resin (Fluka). Resin blocks were crafted, and ultra-thin sections were produced using a Leica Ultracut S. These sections were then placed on glow-discharge Formvar carbon-coated copper grids. Captured images were obtained with a Zeiss LEO 910 electron microscope, which was paired with a TRS Sharpeye CCD camera and the provided software from Tröndle.

### Statistical analysis

Unless specified differently, data are showcased as the average ± s.e.m. The term ‘*N*’ represents biological replicates, meaning individual hearts, whereas ‘*n*’ signifies technical replicates, which could be multiple evaluations on a single fiber or numerous cardiomyocytes from an identical heart. Comprehensive factorial ANOVAs were employed for each response parameter, considering main effects such as genotype (WT and Hom) and treatment (presence or absence of colchicine, Triton X-100, GLN-40 or TEVp treatment). Alternatively, two-sided Student’s *t*-tests were used, pairing them when suitable. If ANOVA model effects were found to be significant, subsequent analyses, such as Tukey’s honestly significant difference (HSD) or Sidak’s comprehensive comparison, were employed to examine differences in group averages. The threshold for significance, alpha, was set at 0.05. Assumptions related to normal distribution and variance homogeneity were assessed with the Shapiro–Wilk normality test, Levene’s variance equality test and residual evaluation. If required to satisfy assumptions, a Box-Cox transformation was applied to the data. In cases where assumptions were not satisfied, a non-parametric approach was adopted, using the Kruskal–Wallis test (a non-parametric version of ANOVA) and Dunn’s method (for non-parametric multiple comparisons). All analyses were performed using GraphPad Prism version 8 software.

### Reporting summary

Further information on research design is available in the Nature Portfolio Reporting Summary linked to this article.

## Supplementary information

Supplementary Tables 1–3.

Reporting Summary

Supplementary Data 1. Source data for Supplementary Tables 1 and 2.

## Source data

Source Data Figs. 1–7 and Extended Data Figs. 1–7 Statistical source data.

Source Data Figs. 1 and 4 and Extended Data Fig. 3 Unprocessed western blots and gels.

## Data Availability

All data pertaining to this work are shown in the text, figures and Supplementary Information.

## Code availability

The code used for force data acquisition on the custom-made setup by Scientific Instruments is available on GitHub (https://github.com/DrDJIng/FiberStretchProgram). The code is customized for this machine but not for the specific experiments conducted in this study.
