# Supplementary material for: Titin governs myocardial passive stiffness with major support from microtubules and actin and the extracellular matrix
Source: Nat Cardiovasc Res. 2023 Oct 26;2(11):991–1002. doi: 10.1038/s44161-023-00348-1 (PMC11358001; doi:10.1038/s44161-023-00348-1)
Supplement: Supplementary file 1 — Supplementary Tables 1–3. [file 44161_2023_348_MOESM1_ESM.pdf]

nature cardiovascular research

Article https://doi.org/10.1038/s44161-023-00348-1

Titin governs myocardial passive stiffness with major support from microtubules and actin and the extracellular matrix

In the format provided by the authors and unedited

Supplementary Tables

Supplementary Table 1. Titin-actin passive force interaction statistics.

**IFs/Desmin passive force contribution mean±SEM (n)**	**Elastic (%)**			**Viscous (%)**		
	Actin severing 1st, titin cleavage 2nd	Titin cleavage 1st, actin severing 2nd	Tukey's mult. comparison (P value)	Actin severing 1st, titin cleavage 2nd	Titin cleavage 1st, actin severing 2nd	Tukey's mult. comparison (P value)
Hom	19 ± 1 (12)	15 ± 1 (11)	0.068	14 ± 2 (12)	8 ± 1 (11)	0.288
**Titin passive force contribution mean±SEM (n)**	**Elastic (%)**			**Viscous (%)**		
	Actin present	Actin severed	2-tailed Student's t-test (P value)	Actin present	Actin severed	2-tailed Students t-test (P value)
Hom	78 ± 2 (11)	73 ± 1 (12)	**0.031**	86 ± 2 (11)	76 ± 4 (12)	**0.026**
Wt	1 ± 2 (7)	0 ± 5 (8)	0.431	13 ± 3 (7)	0 ± 13 (8)	0.362
**Actin passive force contribution mean±SEM (n)**	Titin present	Titin cleaved	2-tailed Student's t-test (P value)	Titin present	Titin cleaved	2-tailed Student's t-test (P value)
Hom	30 ± 3 (12)	28 ± 4 (11)	0.888	38 ± 4 (12)	44 ± 9 (11)	0.522
Wt	35 ± 4 (8)	46 ± 2 (7)	0.054	40 ± 3 (8)	32 ± 6 (7)	0.2148

One-way ANOVA performed on all 4 treatment conditions (Hom+TEVp, Hom+TEVp +TL40, Hom+ TL40, Hom +TL40+ TEVp) followed by Tukey's multiple comparisons test for the statistics specific for the IFs/Desmin contribution.

Supplementary Table 2. Relative contributions of different structural elements to myocardial stiffness.

<table>
<tr><th rowspan="2">Element</th><th colspan="2">Elastic force (% of total)</th><th rowspan="2">Element</th><th colspan="2">Viscous force (% of total)</th></tr>
<tr><td>Low strain (10 %)</td><td>High strain (20 %)</td><td>Low strain (10 %)</td><td>High strain (20 %)</td></tr>
<tr><td>MTs</td><td>21.7</td><td>13.4</td><td>MTs</td><td>34.8</td><td>27.2</td></tr>
<tr><td>Sarcolemma</td><td><0.1</td><td>12.3</td><td>Sarcolemma</td><td><0.1</td><td><0.1</td></tr>
<tr><td>Titin</td><td>54.9</td><td>35.8</td><td>Titin</td><td>28.4</td><td>25.5</td></tr>
<tr><td>Actin</td><td>23.3</td><td>17.9</td><td>Actin</td><td>22.0</td><td>12.4</td></tr>
<tr><td>ECM</td><td rowspan="2"><0.1</td><td>16.7</td><td>ECM</td><td rowspan="2">14.8</td><td>27.8</td></tr>
<tr><td>IFs/desmin</td><td>3.8</td><td>IFs/desmin</td><td>7.1</td></tr>
</table>

Values were determined from fitted experimental curves (Fig. 1e and f, Fig. 2d and e, Fig. 3b and c, and Fig. 4d and f) as well as the isolated cardiomyocyte data (Fig. 5b and j). Sarcolemma after the disruption of the microtubules (MTs, Fig 2d and e) was used to maintain the ‘musical chairs’ approach and as the loss of viscous force in the native left ventricular fiber bundle is likely due to MTs breakdown that cannot be accurately quantified. All data was corrected for preparation type (native/pre-permeabilized) and adjusted to consider tensegrity and experimental error. ECM and IFs (desmin) could not be distinguished from one another at the low strain. ECM, extracellular matrix; IFs, intermediate filaments.

Supplementary Table 3. Antibodies

Target antigen	Vendor or Source	Catalog #	Clone number	Working concentration
Anti-actinin alpha 2, mouse monoclonal	Sigma	Cat. #EA-53	EA-53	IF '(1:100)'
Anti-HaloTag, rabbit polyclonal	Promega	Cat. #G928A	-	IF '(1:100)'
Anti-titin, TTN5 (I20-22), rabbit polyclonal	Myomedix, Mannheim, Germany	Cat. #TTN-5	-	IF '(1:400)'<br>WB '(1:8000)'
anti-α-tubulin, mouse monoclonal	DSHB	Cat. #EA1	EA1	IF '(1:100)'
anti-desmin, mouse monoclonal	Dako	Cat. #Daco M 0760	D33	IF '(1:100)'
TTN-I/A (MIR), rabbit polyclonal	Myomedix	TTN-6	-	WB '(1:20000)'
Cy3 affinipure goat anti-rabbit IgG, goat polyclonal	Jackson ImmunoResearch	Cat. # 111-165-003	-	IF '(1:400)", secondary antibody
Alexa 488 affinipure goat anti-mouse IgG, goat polyclonal	Jackson ImmunoResearch	Cat. # 115-546-146	-	IF '(1:400)", secondary antibody
Alexa 647 affinipure goat anti-rabbit IgG, goat polyclonal	Jackson ImmunoResearch	Cat. # 111-606-047	-	IF '(1:400)", secondary antibody
Anti-rabbit IgG 1.4 nm nanogold, goat polyclonal	Nanoprobes	Cat. # 2003	-	IEM '(1:100)", secondary antibody
Goat IgG anti-rabbit IgG (H+L chain)-HRP	OriGene (Acris)	Cat. #R1364HRP	-	WB '(1:1000)' secondary antibody
