# Supplementary material for: Titin governs myocardial passive stiffness with major support from microtubules and actin and the extracellular matrix
Source: Nat Cardiovasc Res. 2023 Oct 26;2(11):991–1002. doi: 10.1038/s44161-023-00348-1 (PMC11358001; doi:10.1038/s44161-023-00348-1)
Supplement: Supplementary file 5 — Unprocessed western blots and gels. [file 44161_2023_348_MOESM5_ESM.pdf]

Figure 1B

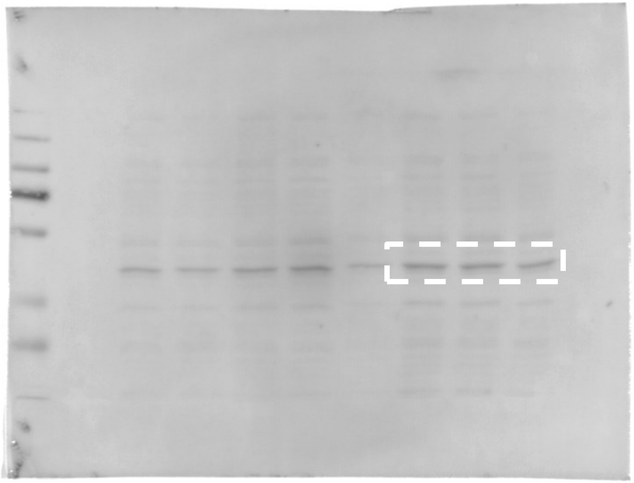

PVDF coomassie stain

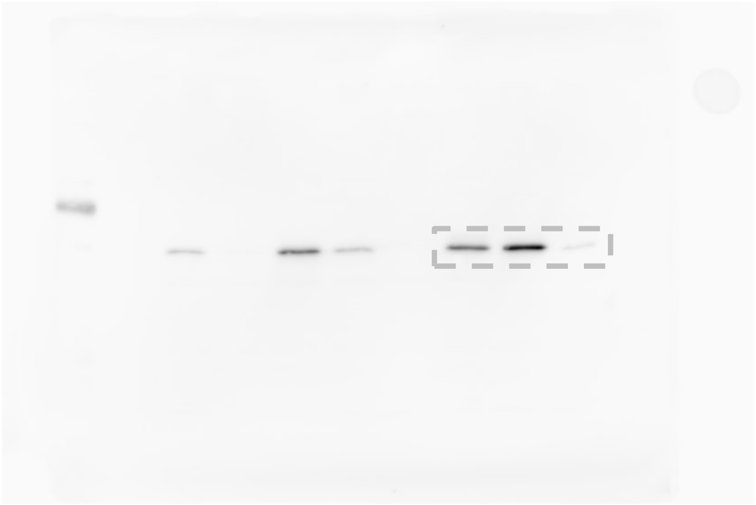

WB: α-tubulin

Figure 4B

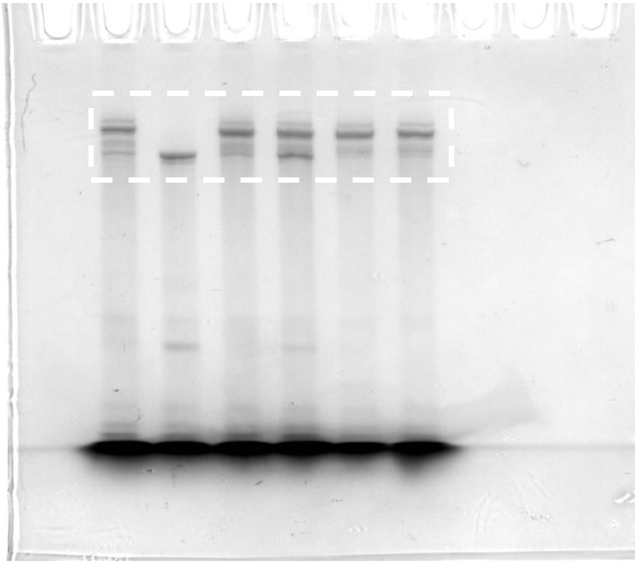

Coomassie stain of titin gel

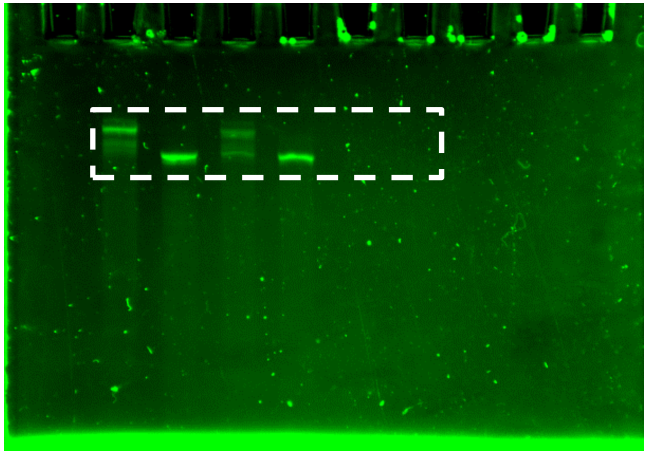

Halotag labelling with HaloLigand-Alexa488

Extended Data Fig. 3C

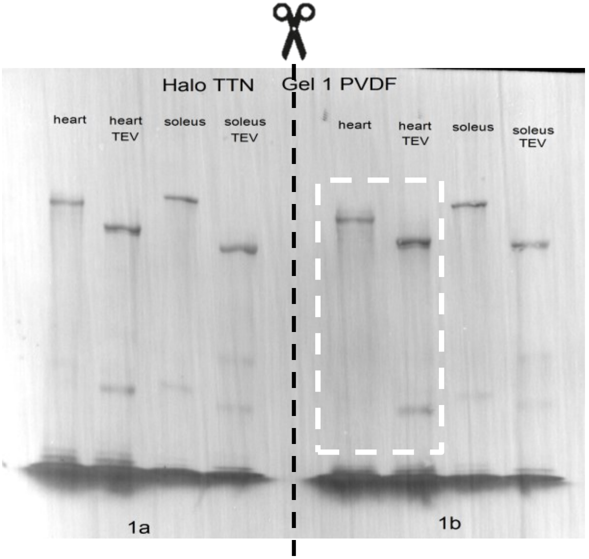

PVDF coomassie stain

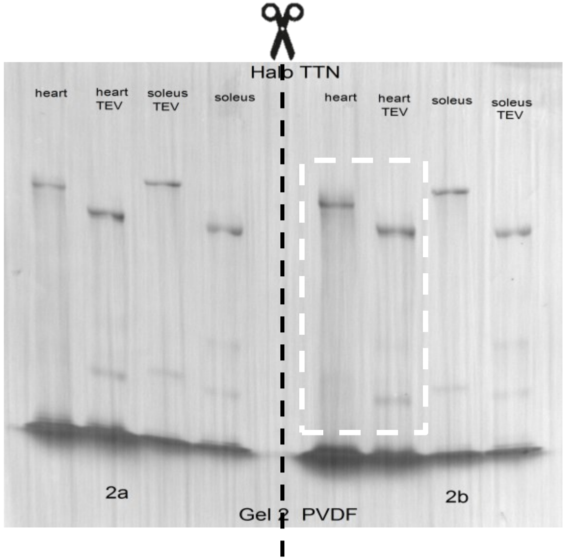

PVDF coomassie stain

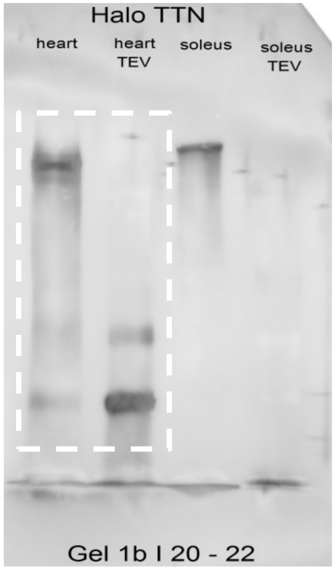

WB: I20-22

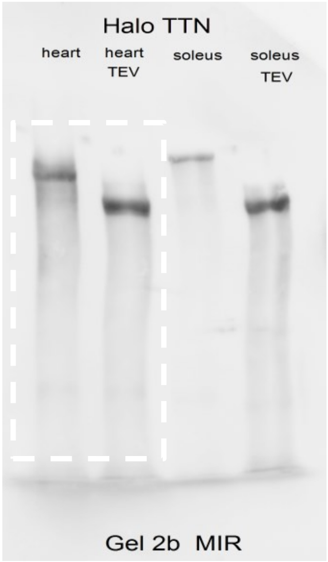

WB: MIR
